# Media Agenda and Press Conferences on COVID-19 in Mexico: An Analysis of Journalists’ Questions

**DOI:** 10.3390/ijerph182212067

**Published:** 2021-11-17

**Authors:** Julio C. Aguila Sánchez, Ninón I. Llano Guibarra, Pamela Pereyra-Zamora

**Affiliations:** 1 Ph.D. Program in Health Science, Faculty of Health Science, University of Alicante, 03690 Alicante, Spain; 2 Faculty of Anthropological Sciences, Autonomous University of Yucatan, 97305 Merida, Mexico; 3 Independent Researcher, 0201 La Paz, Bolivia; nllano@ucol.mx; 4 Department of Community Nursing, Preventive Medicine, Public Health and History of Science, Faculty of Health Science, University of Alicante, 03690 Alicante, Spain; pamela.pereyra@ua.es

**Keywords:** press conferences, COVID-19, media agenda, content analysis, Mexico

## Abstract

The impact of the COVID-19 pandemic has highlighted the need to strengthen health communication in times of crisis. This study aims to analyze the media agenda of press conferences on COVID-19 in Mexico during the first two phases of the pandemic, based on journalists’ questions. The study is based on framing theory. The method used was content analysis from a quantitative perspective. This method was explicitly applied to the final section of the conferences, which dealt with “questions from the press.” The results show that at the beginning of the pandemic, the press was more interested in the government’s management of the health crisis than in issues such as the prevention of the disease itself or the economic impact of the crisis on the country. Moreover, the main characteristic of the questions was that they were generally socially relevant. In conclusion, we found that in the media agenda of the Mexican conference, the frame of attribution of responsibility was prominent but in combination with the frames of conflict, human interest, morality, and economic consequences.

## 1. Introduction

The imprint of the COVID-19 pandemic has made visible the need to strengthen health communication in times of crisis. According to Silva et al. [1], it is vital to deploy effective communication processes to inform about the risks of the health contingency. This process implies communicating but without scaremongering. To this end, it is essential to develop transparent communication with appeals to rationality that clarifies doubts and allows citizens to understand what is happening. Likewise, communication that promotes citizen cooperation in the care of individual and collective health is crucial to overcome the crisis.

These elements highlight the interaction between journalism and public health. In this regard, Bernadas and Llagan [2] point out that COVID-19 made the relationship between journalism and public health visible, which is often overlooked. In order to understand the role of the press in the current crisis, it is necessary to recover the theories of mass communication that explain the place of the press in the (re)creation of social reality. One of these theories is agenda setting [3], according to which the media cannot decide what the masses should think; however, they can impose the issues to think about through their inclusion/exclusion in the public agenda. More than half a century ago, this theory made it clear that the issues discussed or debated in the media become more important than those silenced.

In this respect, framing theory explains that the media frame the ideas and visions of the masses, redirecting their interests to align them with their own. This approach does not mean that the masses are autonomous entities with no capacity to resist media manipulation. In fact, framing theory emphasizes that frames exist not only in the masses but also in the culture where this communicative relationship takes place [4]. In dialogue with this idea, building theory points toward a collective construction of agendas [5]. Hence, it recognizes the bidirectional nature and multiple influences between the different actors in communication [6].

Media communication theories have already been used to analyze media spaces during the current pandemic. According to Syeda [7], journalistic frames and their interpretations would heavily influence media coverage, so a framing perspective is relevant to studying this crisis. In line with this, a study [8] applies framing theory to online news about COVID-19 and finds a repetition of themes about the disease as well as a paucity of narratives about infected or affected people and the discrimination and stigmatization around them.

Similarly, a comparative study of media coverage of the swine flu and the current pandemic in Germany found that at the beginning of both crises, the frames that highlighted responsibility and the effects on the economy prevailed, and later, all frames were used more evenly and had a higher degree of neutrality and objectivity [9]. In another comparative study conducted in four European countries (Germany, Spain, Italy, and the Netherlands), the authors found that radical populist parties emphasized the pandemic less to strategically downplay the severity of the crisis while the number of COVID-19 cases was increasing [10].

In turn, research on news coverage of COVID-19 prevention in Africa claims that the continent was represented and framed differently by Western and Chinese media [11]. According to the author, these media used the language, the tone, the placement of specific facts and arguments to make different judgments in producing African stories [11]. In contrast, and based on a study carried out from the theory of agenda building in Spain, other authors state that the current Prime Minister Pedro Sánchez highlighted main subjects as the political situation, services, and the conflict scenario around coronavirus [12]. Similarly, the Chinese newspaper Global Times highlights the difficulty of building a participatory media agenda in a communication and political context as authoritarian as China’s [13].

Another study [14] notes that risk communication and the government’s emergency response encouraged citizen participation in dealing with the virus in China. This resulted in reduced anxiety in society and increased public confidence in the government. The authors agree that the decrease in infections was due to the country’s risk communication, prevention, and control measures. However, they acknowledge an information bombardment during the pandemic that interfered with the communication effectiveness [14].

These communication theories have also been used to describe how the pandemic has swamped media coverage, crowding out other important issues. For example, a Finnish newspaper study shows how the beginning phase of the pandemic in 2020 was related to a drop in previous climate coverage [15]. These studies demonstrate the applicability of communication theories in research on media coverage of COVID-19. However, they usually analyze print or digital media, including social networks such as Twitter, but rarely include the media agenda in official communication spaces such as press conferences.

Consequently, there are currently few studies on the role of the press in press conferences on COVID-19 in Mexico. Therefore, this article sets out to analyze the media agenda of press conferences on COVID-19 in Mexico during the first two phases of the pandemic based on journalists’ questions. This objective derived from the following research question: What has characterized journalists’ questions at press conferences on COVID-19 in Mexico during the first two pandemic phases?

The press conferences have been attended by journalists from the country’s leading media outlets. According to experts [16], there is a peculiar relationship between the current Mexican government and the country’s media. This connection is related to the predominant ideologies in these media, which determine the positions of opposition or assimilation of officialdom. For example, Villegas states, “La Jornada, Reforma, and Milenio Diario are depicted as representing left, right and center ideological politics, respectively,” and all three are among the newspapers with the largest audiences in the country ([17], pp. 37–38).

The current president has openly described some media outlets as “representatives of interests contrary to his project” ([17], p. 17). These include Reforma, El Financiero, Milenio, El Universal, Grupo Fórmula, Excelsior, Milenio, and Grupo Multimedios, among others [16,18,19]. Other authors also agree that Excélsior has always been characterized by its conservative stance [19]. At the other extreme are newspapers such as La Jornada, of the center-left [18], or Canal Once, which, according to Pareja, has been a more progressive media [20].

An illustrative fact about the relationship between the current government and the media is that in 2014, a newspaper such as Milenio, under the previous government, billed 48 million Mexican pesos in official advertising, while La Jornada billed 59 million. On the other hand, in 2019, with the current government, La Jornada invoiced 252 million and Milenio invoiced only 67 million [20]. This figure shows how relations with the traditionally privileged media have been reversed in the current legislature.

It is worth noting that Mexico is one of the hardest-hit countries in the Latin American region, with one of the highest mortality rates in the world [21]. On the other hand, this study is about a pioneering space in the Latin American region, such as the Mexican government’s press conference to address the pandemic. This space had almost daily broadcasts, both on national television and on various digital platforms. Despite this, there are very few studies on this official communication space of the Mexican government during the coronavirus crisis.

## 2. Materials and Methods

### 2.1. Method

The content analysis method [22] was used for the study from a quantitative perspective. It was applied to the Mexican government’s press conferences on COVID-19, specifically to the “questions from the press” in the final section of the conference, to analyze the press’s characteristics, themes, interests, and intentions.

### 2.2. Press Conferences as Sources of Information

The Mexican government’s press conferences on COVID-19 began on 29 February 2020, when the global health emergency started. These conferences were aimed at reporting on the evolution of the pandemic in Mexico and, from the beginning, have been organized and chaired by officials from the Health Secretariat, with the participation of other institutions.

The conferences have been held daily and have lasted approximately one hour each. They have been broadcast live on television and streamed on social media (at 7 pm), with a large audience [23]. To this day, all the conferences are archived on the YouTube channel and on the website of the Health Secretariat, which has facilitated their study. The conferences had a clear agenda. They usually began with a presentation of data on the evolution of the disease at the international and national levels. This part was followed by a specific topic, ranging from the recruitment of health workers to new scientific findings on the pandemic. In the end, the press had a space for questions to the speakers. This last space is the focus of the study.

### 2.3. Population

This study analyzed journalists’ questions to the speakers during phase 1 (29 February to 23 March) and phase 2 (24 March to 20 April) of the press conference. There were a total of 51 conferences (23 and 28 respectively). A total of 738 questions were recorded (see Supplementary S1) in 304 interventions from 102 journalists. All conferences were viewed in their entirety in order to contextualize the journalists’ questions. This material amounted to around 60 h of viewing. This shortening of phases 1 and 2 was because there was a context of uncertainty and little planning at the beginning of the pandemic. In this period, the concerns and interests of society and the press on the subject could be expressed more naturally.

Information about the attending media was requested from the Government of Mexico’s Transparency Platform to contrast their data with our results. This request was possible because such information is considered to be in the public domain.

### 2.4. Participating Media: Their Characteristics

In the analysis, we included all media outlets whose journalists asked questions at the press conferences. A total of 1030 journalists from 134 media outlets attended. It is worth noting that in particular cases (17), journalists did not register their media outlet. In summary, we worked with the 102 journalists who asked questions at the conferences and with the 57 identified media outlets to which they belonged. Table 1 below shows the characteristics (type of organization and political tendency) of the 15 media outlets to which the journalists who asked the most questions at the press conferences belonged.

### 2.5. Instrument and Study Variables

The analysis sheet was designed with three sections to collect: (1) data from the conference, (2) data from the journalists, and (3) data on the questions. The data were processed through the File Maker 8 program (Claris International, Massachusetts, the United States of America), in which a record card was constructed to transcribe and organize the information according to the variables previously defined. The data were entered manually into the File Maker program as the lectures were viewed. The viewing was done with subtitles so as not to lose any detail. The categorization of the information is based on a previous study on these press conferences [24]. Two independent researchers made the categorization of the questions. A third reviewer discussed and resolved differences. The authors used the Kappa index to assess the concordance of the classification (k = 0.98), finding almost perfect concordance between researchers.

The database was exported from File Maker 8 (Claris International, Massachusetts, the United States of America) to Excel^®^ (Microsoft, Redmond, WA, USA), and SPSS v.26^®^ (IBM Corporation, Chicago, IL, USA), for statistical analysis. Percentages and cross-tabulations were calculated for the various variables described in Table 2.

## 3. Results

### 3.1. Journalists’ Questions: What Issues Make Up Their Agendas?

When analyzing the 738 questions recorded, questions were found that corresponded to the seven themes foreseen in the instrument. The most frequent were questions on government management of the crisis. These included the following questions: “On procurement, how would you operate, would the states do it?” or “The US and Canada have already declared a state of emergency, you have told us this is not an emergency, have you not considered changing your strategy?” Questions about the statistics of the disease also stood out, such as: “Have the cases in Jalapa, Veracruz been confirmed?” or “Are the 39 suspects you were talking about already on the table?” This was followed by questions on communication and health education for the population, with questions such as: “About pregnant women, from what stage should they take care of themselves?” or “About the elderly, what should their daily routine be like?”.

There were also questions about the characteristics of the disease itself: “What happens if a person is infected with the two strains that so far exist in China?” or “People lose their sense of smell and taste, are the symptoms exclusively those we have been told or have new ones been discovered?” Likewise, there are fewer questions that deal with politics and officialdom, such as the following: “In the networks, they are saying: ‘Dr. López-Gatell was forced to reveal the truth,’ I would like to ask him to clarify this situation again” or “The governor of Hidalgo tested positive, he was with Andrés Manuel in La Mañanera, is he going to take the test, or is he going to take isolation measures?”

**Figure 1:**
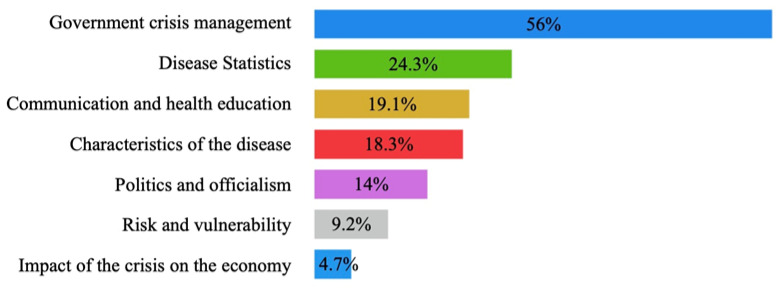
Percentages of the topics of the journalists’ questions.

Likewise, among the questions on risk and vulnerability are the following: “Regarding materials in other indigenous languages (Nahuatl, Mayan) […], what is going to be done?” or “What would be the strategy suggested to the states for people in street situations?” Moreover, finally, there are questions about the impact of the crisis on the economy: “How do the new measures affect the private and public sector?” or “How could companies act, can they lower wages after today’s measures?” The Figure 1 shows the percentages for each of the question topics.

**Figure 2:**
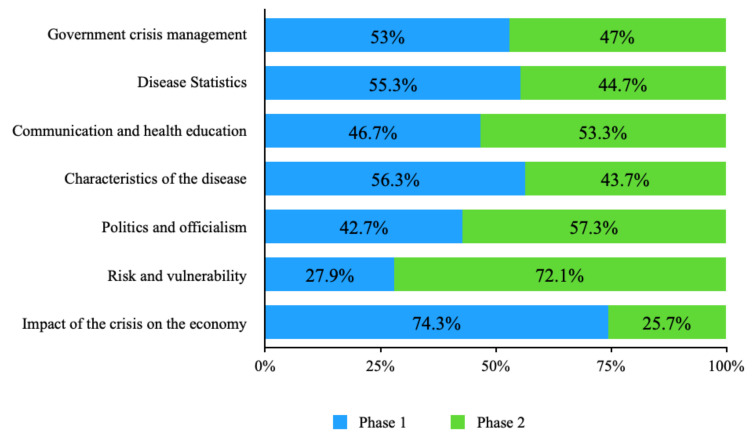
Percentage distribution of questions in phases 1 and 2.

When analyzing the themes of the questions, it was observed that of the total number of questions on the impact of the crisis on the economy, 74.3% were asked in phase 1, and only 25.7% were asked in phase 2. In general, few questions addressed this issue: only 35 out of 738 questions were asked. In contrast, concerns about risk and vulnerability increased as the conferences progressed: only 27.9% were asked in phase 1, while phase 2 accounted for 72.1% of the total number of questions. The rest of the issues identified behaved similarly in both phases, with no significant percentage difference. These percentages are represented in Figure 2.

On the other hand, interest in governmental management of the crisis was also prominent in all media. The most notable of these were questions on the statistics of the disease in the following media: Reforma (38.6%), La Jornada (37.9%), Milenio (28.6%), and Grupo Imagen (27.8%); while questions on communication and health education were the most notable in Uno TV (34.6%), La Jornada (29.3%), and Plenilunia (25%). Similarly, questions about the characteristics of the disease stood out among Excelsior (20%) and Plenilunia (18.2%). In contrast, questions about politics and officialdom were more frequent in MVS Noticias (38.9%) and Notimex TV (19.2%).

### 3.2. Journalists’ Questions: Main Characteristics

Of the 738 questions registered, more than half (54.5%) had social relevance in that they clarified doubts or dealt with issues of public interest. In turn, almost a quarter of the questions were related to that day’s conference (24%) and an event in the last 24 h (24.1%). An example of the latter was when a journalist asked the Undersecretary of Health, Hugo López-Gatell, to respond to a negative comment about his work: “Yesterday […], a TV host was talking about you, precisely about not paying attention to you as a health authority, can you give us your point of view?”

Sensationalist overtones were also found in some questions (18.2%), such as: “Can you give us an estimate of how many deaths there will be, tens, thousands or millions? I mean to reassure people”. In addition, another day, a journalist urgently wanted to know if a patient had died recently, when perhaps not even the family knew: “Now that it is 7:38, […] with all due respect if you can confirm before the end of the conference if the person died?”

Some questions became repetitive (21.8%), especially on topics such as the sentinel model adopted by the country and the purchase and management of supplies, or statistics, which could be less comprehensible at the beginning of the pandemic. Questions addressed to other secretariats (8.3%) were the least common because most of the questions were addressed to officials of the Health Secretariat, which was the institution organizing the conference, and whose officials attended the conference daily.

**Figure 3:**
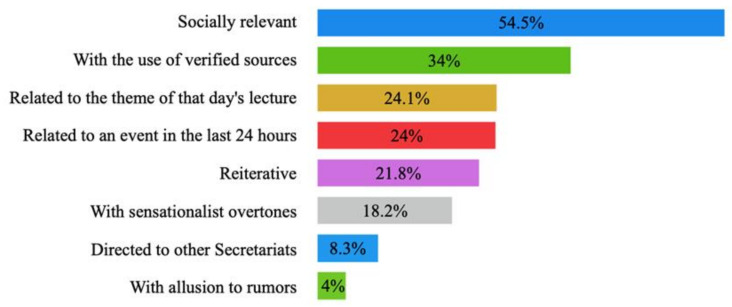
Percentage distribution of the characteristics of the journalists’ questions.

In terms of other data on journalistic work, one-third of the questions asked used verified sources (34%) to argue them or give them greater credibility. Very few questions alluded to rumors (4%), either clarifying them or giving them a voice. An illustrative example was when a journalist asked: “Milenio talks about a hospital in fear of the virus. Can you corroborate if it is true that it takes five days for patients to get their test results?” These percentages are represented in Figure 3.

When analyzing the characteristics of the questions concerning other variables of the study, we found that at the beginning, questions related to an event in the last 24 h were more frequent: 59.6% of the questions were asked in phase 1, and 40.4% were asked in phase 2. Reiterative questions were also more frequent in the initial phase: 71.4% of the total corresponding to phase 1 and only 28.6% corresponded to phase 2. As time went on, journalists diversified their questions and topics.

Among the characteristics of the questions, it has been observed that in all the media, questions with social relevance stood out, except in the cases of repetitive questions, which were more common in Grupo Imagen (77.8%) and those related to some event in the last 24 h in MVS Noticias (55.6%). Following these, the most notable were those related to the theme of that day’s conference, on Uno TV (34.6%), La Jornada (32.8%), and Reforma (30%), while those related to an event in the last 24 h were the second most notable on Canal Once (34.1%), Milenio (33.7%), and Notimex TV (30.8%). Moreover, questions with sensationalist overtones were most common on Excelsior (48%).

**Figure 4:**
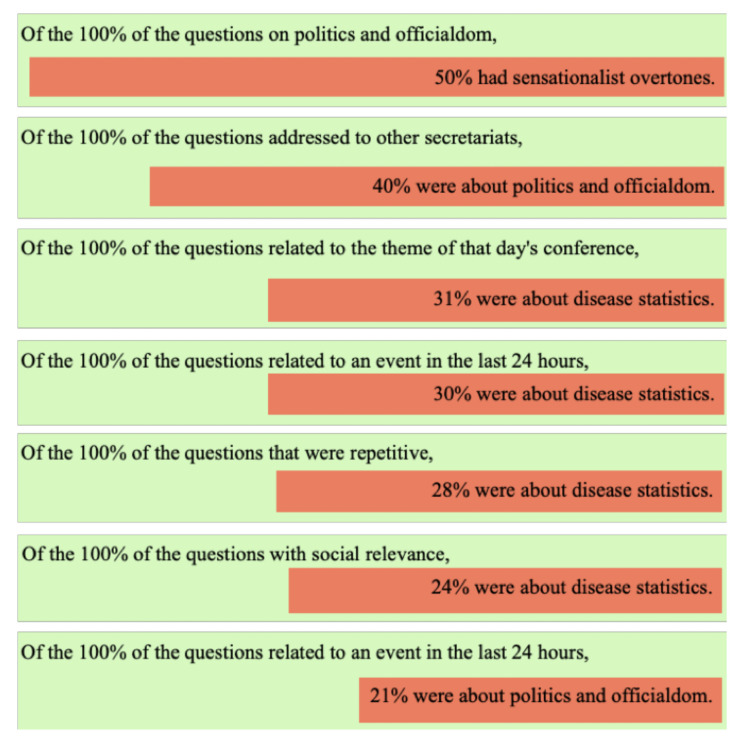
Crossovers between questions characteristics and questions topics.

When we analyzed the themes and characteristics of the questions together, we found that 50% of the questions on politics and officialdom had sensationalist overtones. In addition, 40% of the questions were addressed to other secretariats, and 21% of the questions related to an event in the last 24 h were about politics and officialdom. On the other hand, questions about disease statistics accounted for 31% of those related to the theme of that day’s conference, 30% of those related to an event in the last 24 h, 28% of those that were repetitive, and 24% of those with social relevance. These percentages are represented in Figure 4.

## 4. Discussion

At the beginning of the conferences, economic issues were more present in the journalists’ questions, demonstrating the uncertainty about the impact of the health crisis on the economy. In addition, the progressive reduction of questions on the impact of the crisis on the economy and the increase in questions on risk and vulnerability shows the shift of concerns toward the impact of the health and economic crisis on specific groups, such as essential workers, the unemployed, dependents, the homeless, or women.

In terms of media performance, it is interesting that La Jornada, Uno TV, Excelsior, Grupo Imagen, and MVS Noticias were among the ten media outlets whose journalists asked the most questions in general; and at the same time, none of their questions were about the impact of the crisis on the economy. In turn, these data are very illustrative of the sensitivity of the mainstream media to the consequences of the health crisis on the most affected sectors of the population. In contrast, Plenilunia, a small, independent media outlet, is among the most interested in questions about the characteristics of the disease itself. Finally, the fact that questions with sensationalist overtones are the most common among Excelsior journalists is a sign of how they construct the media agenda during a health crisis, regardless of the consequences. Let us remember that Excelsior, since its beginnings, has been a newspaper characterized by its conservative stance [19]).

In addition, the fact that at the beginning, the questions were more related to some event in the last 24 h is an example of the spontaneity in the construction of the agenda, as a reflection of the generalized uncertainty and, in this framework, the relevance of any event. The same goes for repetitive questions (which were more frequent at the beginning of the pandemic). As a subject that journalists had little knowledge of, there were more doubts at the beginning and precisely because of its novelty. In addition, the concomitance between questions about politics and officialdom and questions with sensationalist overtones could point to a close relationship between these two types of questions.

These results illustrate the role of the press in the management of the coronavirus crisis in Mexico and elsewhere in the world. Heras-Pedrosa et al. [25] point out the decisive role that risk communication plays in informing, transmitting, and channeling the flow of information in society. Another study focused on the influence of communication variables in the generation of judgments about the possible personal consequences of the coronavirus pandemic [26] and the management of the uncertainty and fear generated in the population by a crisis of such magnitude. Hence the importance of being careful with the topics included in the media agenda and, above all, with the approaches—more or less alarmist—from which they are framed [27]. It has been shown that the high degree of politicization in the initial coverage of COVID-19 may have contributed to the polarization in the attitudes of the virus in the US, which highlights and complicates the importance of the role of the press in the current crisis [28].

The thematic classification of the journalists’ questions shows that of the five frames proposed by the framing theory [29]—the attribution of responsibility, conflict, human interest, morality, and economic consequences—the attribution of responsibility was more prominent in this case. In other words, journalists were more concerned with questioning those responsible for managing the crisis. This scenario is not new; for some years now, it has been discovered that it is common for the media to focus on this frame [30]. A previous study in Mexico and Spain noted that the use of frames showed the definition of Swine Flu as an event with serious consequences for citizens, where the leaders must assume responsibility for dealing with the problem [31].

The themes favored in the analyzed questions coincide with those prioritized in other media on COVID-19 in other countries. In a reflection on the pandemic, Balog-Way and McComas [32] identified “three key risk communication messages: (1) staying at home, (2) some groups are at higher risk, and (3) daily infections and deaths (p. 1).” These three lines are found in the journalists’ questions in this study. However, although journalists in Mexico ask less about economic issues, this is a persistent topic in other contexts. Similarly, in a study analyzing the foreign press in Spain, “778 news items on COVID-19 were identified, with a prevalence in the US press and mainly on economic rather than health issues” ([33], p. 1).

The journalists’ questions also placed the issues of risk and vulnerability associated with COVID-19 in Mexico on the media and public agenda. However, it was a topic that was dealt with to a lesser extent than others, such as government management of the crisis or statistics on the disease. In this regard, a comparative public good communication study between Argentina and Spain highlights that it has not yet been generated the space for the economic, social, and psychological consequences that COVID-19 is causing: increased poverty and gender violence, depression, anxiety, worry, uncertainty [34].

Segura [35] refers to this as a prevention campaign with a privileged target in the middle and upper classes, which is a class bias in the coverage because it reproduces the governmental messages of staying at home without taking into consideration the conditions in which the less privileged classes live and work. According to the author [35], in this way, in Argentina is ignored or unknown “what happens to a third of the country’s population who live in poverty and do not have these living conditions” (p. 57).

## 5. Conclusions

In conclusion, the journalists’ questions were more focused on the government’s management of the health crisis, and to a lesser extent, on the disease itself or the political and economic repercussions of the health crisis in the country. Similarly, the press played a significant role at the beginning of the pandemic by putting public, media, and political interest issues on the public agenda. However, the media agenda of the press conferences has reflected the spontaneity with which the situation was handled, which was typical of crisis contexts. These data are also indicators of the lags in health communication compared to other areas of communication.

However, the data analysis made it possible to achieve the proposed objective of characterizing the media agenda of the press in the Mexican government’s press conferences on COVID-19 in the first two phases. One of the main contributions of this characterization is that it provides data on the limits and strengths of the Mexican press’ actions in the face of the COVID-19 crisis. These data allow us to understand how the press has contributed to, and at the same time hindered, the overcoming of the crisis. These results also serve to review what has been done, learn from it, and improve future action.

This work also helps identify the extent to which the media influence the formation of Mexican public opinion in the face of the COVID-19 crisis, especially in its early stages. The data show that the media did not question the press conference as a space for official communication. Instead, they used it to attack the government, sometimes to misinform and make political noise, rather than help the conference fulfill its objective of informing and debating the disease and its economic impact.

The government’s performance at the conferences also contributed to this situation by using it for its legitimization. In other words, both the Mexican government and the journalists used the space to pursue their information interests. In this way, the crucial issues of the disease were not prioritized either by the media or by those in power, thus affecting the processes of communication and health education of the population.

An example of this distortion of issues occurred with the debate on the appropriateness of using masks, which was not exclusive to Mexico but occurred internationally. In that case, instead of reporting on the ideas contained in the controversy, the Mexican media defended its use, and others did not attack the opinion of certain politicians or officials. This is the case of the Undersecretary of Health, who argued that “its use was not scientifically proven to be effective.” In general, the media turned to attacking him rather than contradicting him, but at the same time reporting the importance of using it for health care as other specialists were recommending.

Instead, in these cases, the focus was on attacking and sensationalizing public officials’ headlines because that attracted more public attention. In this way, the media was more about attacking the politician than verifying or contrasting information and pointing blame rather than reporting or at least questioning or scrutinizing communication and health policies.

In turn, this idea justifies the need to develop training plans for media communication professionals on information, education, and communication for health. As this study demonstrates, journalists or communicators play a fundamental role in these cases, as they are the ones who have the possibility of bringing the state’s actions into the debate and at the same time giving voice to the concerns and needs of the population.

In addition, the study’s main limitation is that it only covered the first two phases of the pandemic. Thus, phase 3 and the new normal were left out of the study. In this sense, work is recommended to continue the missing phases to complete the conferences’ systematization. This effort would make it possible to identify the regularities that characterize the role of the Mexican press, not only at the beginning of the pandemic but also up to the present day, and even to make a comparison from beginning to end once the crisis has been overcome.

## Figures and Tables

**Table 1:** Type of organization and the political tendency of the 15 participating media outlets.

Media	Number of Questions	Type of Organization	Political Tendency
Milenio	98	Private	More in opposition
Reforma	70	Private	More in opposition
La Jornada	58	Public	More officialist
Canal Once	41	Public	More officialist
Notimex TV	26	Public	More officialist
Uno TV	26	Private	More in opposition
Excelsior	25	Private	More in opposition
Plenilunia	22	Private	More neutral
Grupo Imagen	18	Private	More in opposition
MVS Noticias	18	Private	More in opposition
Grupo Fórmula	17	Private	More in opposition
Eje Central	16	Private	More in opposition
Medicina digital	16	Private	More neutral
Periódico Bajo Palabra	14	Private	More neutral
Multimedios Televisión	13	Private	More in opposition

**Table 2:** Variables analyzed in the study.

Categories			Content of Variables
Registration		Conferences	Phase, date, topic, duration, and number of journalists
		Journalists	Gender, number of questions, and medium of communication
On journalists’ questions	Themes of the questions	Government crisis management	On government actions to address the crisis
		Disease statistics	About COVID-19 infections and deaths
		Characteristics of the disease	On the actual characteristics of COVID-19 as a disease
		Politics and officialism	On political opinions for or against the Mexican government
		Risk and vulnerability	On socio-economic situations that increase exposure to risk
		Communication and health education	On health care and raising awareness of the crisis in the population
		Impact of the crisis on the economy	On the impact of the crisis on the economy at both the national and individual level
	Question characteristics	Socially relevant	They deal with an issue of public interest
		With the use of verified sources	They cite a source that can be corroborated
		Related to the theme of that day’s lecture	They are aimed at clarifying the central theme to which the conference is dedicated
		Related to an event in the last 24 h	They seek to clarify some recent event, even if it is outside the framework of the conference but is of interest to the press and the population
		With sensationalist overtones	They inquire into elements of private life, contain emotional elements, and seek to generate controversy and alarmism
		Reiterative	They intend to clarify previous answers or keep the topic under discussion
		Directed to other secretariats	They are not addressed to the officials of the Health Secretariat but others
		With allusion to rumors	They quote some unverifiable source

## Data Availability

Data are available at https://coronavirus.gob.mx/noticias/ (accessed on 9 November 2021).

## Supplementary Materials

The following are available online at https://www.mdpi.com/article/10.3390/ijerph182212067/s1, Supplementary S1: Journalists’ questions.
