# Supplementary material for: Media Agenda and Press Conferences on COVID-19 in Mexico: An Analysis of Journalists’ Questions
Source: Int J Environ Res Public Health. 2021 Nov 17;18(22):12067. doi: 10.3390/ijerph182212067 (PMC8625423; doi:10.3390/ijerph182212067)
Supplement: Supplementary file 1 [file ijerph-18-12067-s001.zip › ijerph-1402491-supplementary.pdf]

International Journal of
Environmental Research and Public Health

Supplementary S1. Journalists' questions.

Phase 1:

	Question	Media
1	Regarding the detection and diagnosis processes, if in the 32 states these spaces really exist, they somehow certify this type of diagnostic detection.	A tiempo TV
2	In the case of Torreón, the detection had taken place in a private institution. How should private institutions behave in this regard?	A tiempo TV
3	Is AMLO in the risk groups? Because of the age	Periódico digital Bajo Palabra
4	What precautions would you recommend to Mr. President?	Periódico digital Bajo Palabra
5	Do you think that the president should take the same precautions as the rest of the citizenry?	Periódico digital Bajo Palabra
6	Of the case that he explained to us, he tells us that there were four positives and one carrier. So they are not cataloging it as a confirmed or positive case?	La Jornada
7	So, is it possible that at this time, there are many more people in Mexico who may already have the virus but are asymptomatic?	La Jornada
8	When they tell us about this network of 31 public laboratories that can carry out the tests, they are still sending INDRE for a second confirmation, until when will it be like this?	La Jornada
9	So do all the cases come from Italy?	La Jornada
10	What is the coronavirus test?	Medicina digital
11	How many of the laboratories are certified?	Medicina digital
12	There is a department that monitors fake news, how many people work there?	Eje Central
13	Is that department made expressly for the crisis on the internet?	Eje Central
14	Was there or is there a budget for this team?	Eje Central
15	Why do cases have to be confidential, and we cannot know the names of the cases?	Eje Central
16	Will there be a protocol to unify the information of the states?	Milenio
17	In what situation is the young interned at the INEI?	Milenio
18	How many are the suspected cases in details by states?	Milenio
19	Does the case of Sinaloa that was a companion and had the same symptoms as that of Mexico, do you already have the results of that test?	Reforma
20	Is it confirmed in San Luis Potosí as the governor said?	Reforma
21	How long will the case be held?	Reforma
22	The family is good?	Reforma
23	Is this person from the INEI true that he could have had contact with hundreds of people in the restaurant of his property?	Reforma
24	Are all the contacts controlled?	Reforma
25	Is it true of an alleged case that today arrived at the INER on its own feet and escaped from the institute?	Reforma
26	These two specific cases of CDMX, what is the medical part in addition to mild symptoms	TV Azteca

27	Both in the INER and the national institute of nutrition, if extra measures are to be taken in the hospital with the people who go to see their relatives?	TV Azteca
28	So, isn't there a special protocol?	TV Azteca
29	A death occurred in a Pemex hospital in Villahermosa Tabasco. Does this have to do with the coronavirus?	No identificado
30	In the case of influenza, it is better not to take medicine for fever, and in this case, it is possible. Does it mean that it is now milder?	No identificado
31	You have reported that at this time, those infected by the coronavirus have been imported and that there are only 5 people with the case of Chiapas, and you have also said that with 100 cases, it would be community transmission. When could this stage be reached?	Periódico digital Bajo Palabra
32	How could 5 of could go to 100	Periódico digital Bajo Palabra
33	Do we risk excess confidence in Mexico in the face of the coronavirus?	Periódico digital Bajo Palabra
34	Are you calculating that in 40 days there will be community dispersal? What would it depend on?	Reforma
35	There are 11 suspected cases in which states and how are they?	Reforma
36	Could 161,000 cases occur as when influenza?	Reforma
37	According to the data, 78 tests have been done on people who meet the profile. Could you repeat the profile?	Medicina digital
38	Wouldn't we expect the number of cases to be higher due to the trafficking of people traveling from these countries?	Medicina digital
39	They are reporting to me that what report they have of two suspected cases, a man and a woman in the towns of Posa Rica, Jalapa.	Vanguardia Veracruz
40	In the afternoon, the coordinator of Morena said that he is going to request that a fund of 25 million pesos be approved to address the coronavirus situation in Mexico. How is this situation because you said that greater resources were not needed?	Heraldo de México
41	What protocol have been installed in the airports?	Canal once
42	The INER patient is discharged, what has happened to the other patients?	MVS
43	Of the contacts, how many of these 5 patients in total have they already tracked? I understand 8 were missing. Know if only relatives or there are others	Milenio
44	Also if the suspects are possibly 2nd generation infections?	Milenio
45	Is there a contact missing? Who are they? Someone who met at the airport for example?	Milenio
46	Regarding the genome, how important is how the virus is composed for Mexico?	La Jornada
47	Regarding the treatments, could you specify the ARV trials of HIV patients, and is there another one produced in Cuba that has possibilities?	La Jornada
48	Regarding the case of San Antonio, Texas, is there an alert for the proximity or flow of Mexicans in San Antonio?	Reforma
49	And about the case in Edomex, now they talked about a couple, could you talk about that?	Reforma
50	How long does the virus last on the surface?	Uno TV
51	These 2 clinical trials with chloroquin and remdesivir, do they exist in Mexico?	Uno TV
52	Would these trials be given in hospitalized patients?	Uno TV

53	The PRD deputy rejects what Gatell had commented and makes a comment calling positive cases contaminated. Any reaction to this?	Uno TV
54	What is the protocol at the southern border of Mexico?	Eje Central
55	So there are no filters at airports?	Eje Central
56	Will we already have an estimate of infection rates for the Mexican case?	Grupo fórmula
57	In the case of the discharged patient, what is the post hospital process?	Grupo fórmula
58	In China there are cases that are contagious again, can that happen?	Grupo fórmula
59	A piece of news has come out that materials are missing. What can you say about it?	IMER Noticias
60	If the virus grows, will there be special hospitals like in Wuhan?	IMER Noticias
61	Did you already have any other hospital north of the city to receive a case, or will they be opened as it progresses?	No identificado
62	The doctors of the XXI century national medical center report that they had no material. Do you know of this?	No identificado
63	Why in the last 24 hours no case has been confirmed when it does increase this from 39 suspects	MVS
64	What are the 4 entities that do not report suspicious cases?	MVS
65	And concerning the 44-year-old person in the general hospital in Mexico, have they already had the test, and it came out positive, and are you waiting for confirmation?	MVS
66	They have already evaluated how many resources they would require because they had rejected the sum of 25 million that the governor spoke.	Milenio
67	Are there returnees among the suspects?	Milenio
68	Who was the supplier of heparin in the case of Pemex? And if it was an administration purchase error	Milenio
69	Where do the suspected cases come from?	Milenio
70	The UN made a call to Latin America, given that there are few cases, about making stronger measures. What to answer to this?	Uno TV
71	Tomorrow you meet with governors. What is the objective of that meeting?	Uno TV
72	Do the projections correspond to what has happened?	Canal once
73	Today the agency reported on the visit of the Italian ambassador, the meeting was held and the issue of the coronavirus was discussed, I would like to know in what tenor the meeting was, what they talked about the subject.	Canal once
74	Is the woman who is hospitalized isolated, mild or severe?	Canal once
75	What is known about almost measles in a girl? The family says that the girl was not vaccinated	Grupo ACIR
76	You can tell us if we are already in scenario 2?	Reforma
77	With what other embassy have you had a communication to talk about coronavirus?	Reforma
78	INER staff are afraid of not having supplies, is there a plan to solve this?	Reforma
79	What is the capacity of fans installed in the INER and how many are in use?	Reforma
80	If there were an escalation of cases, are they anticipating the infrastructure deficiencies that Mexico has?	Medicina digital
81	Can entities confirm the cases? How do we know that the information is real?	Radio Centro

82	Of the 39 suspects who spoke are already in the table?	TV Azteca
83	How are the states? Are you going to need the supplies?	Excélsior
84	Are you in favor of the budget?	Excélsior
85	About Pemex, to know what happened in the hospital, who is the supplier of heparin sodium?	Excélsior
86	What is known about the measles case?	Excélsior
87	In that meeting, they spoke if you agree that we are in this first phase right now? What is the vision of the governors?	MVS
88	Alfonso Romo said that we are in an emergency, contrary to what Gatell said, and pointed out that the release of resources does not have to be bureaucratic	MVS
89	Specific information campaigns would be carried out in this first phase for schools and universities, or it would be to the general population.	MVS
90	It has been said that Mexico has only presented contagions by importation, that is why roads or airports will not be closed ... what would be the orientation and message to internal and international tourists?	Periódico digital Bajo Palabra
91	What measures are being implemented to better monitor food and restaurant production?	Radio Trece
92	Regarding the international collaboration of Mexico in the region	Radio Trece
93	Today the death of a man was registered on a cruise ship in California, and they declared a state of emergency, is there any special plan for the border with the United States?	TV Azteca
94	A cruise ship did not dock in Ensenada, is there a risk for Mexico?	TV Azteca
95	The agency notified that there are 480 professionals trained for the coronavirus. I would like to know the profile of these people.	Canal once
96	Are first contact doctors contemplated?	Canal once
97	Is there anyone on cruise ships who can identify a patient with coronavirus?	Canal once
98	If the states are going to do tests, who will continue with the stewardship of the reports? How is it going to be?	Canal once
99	Governor, can you tell me what measures the state representatives will take in the short term?	Canal once
100	About the supplies, they say that they are complete in scenario 1, but what are they and also in scenario 3?	Reforma
101	Gatell commented that the reserve is in very bad condition. Could you comment?	Reforma
102	The cases of Jalapa, Veracruz were confirmed?	Vanguardia Veracruz
103	In scenario 3, how many people can become ill?	El Universal
104	In that sense, how much resources do we need?	El Universal
105	How much has the sale of face masks increased in the country?	El Universal
106	How is the training for pharmacy doctors going?	No identificado
107	measles happened in which municipalities?	No identificado
108	What happens with the 6 patients from the Pemex hospital to one from the CDMX?	No identificado
109	There are currently supplies for 300 thousand cases?	Canal once
110	Are there enough resources for a scenario 2?	Canal once
111	They already have information about the cruise that came from the United States and had made a tour in Mexico. Did it represent a risk to the country?	Heraldo de México

112	What is the amount that has been disposed of in Mexico for the contingency?	Eje Central
113	Specify a bit if the Senate figure of 20 million pesos is necessary?	Eje Central
114	How much has been spent until these days?	Eje Central
115	Of the strategic reserve that you have right now, what percentage of scenario 3 covers?	Uno TV
116	These 300 cases that we talked about in scenario 3, would they be serious or minor?	Notimex TV
117	Do you have the estimate of the purchase of mouth covers, and how does it affect the doctors?	Notimex TV
118	How much hope can there be from the second week of April that influenza levels will surely drop in the world?	Medicina digital
119	On the statements of the former health secretary regarding the risk of the southeast?	Medicina digital
120	How do you see the United States and Belize putting restrictions on passengers from China?	Medicina digital
121	When will we get to stage 3?	Milenio
122	Where are these inputs to be purchased? Are they produced in Mexico?	Milenio
123	What type of medications is it?	Milenio
124	Regarding measles, does Mexico have the necessary vaccines insured?	Milenio
125	Where is the 6th case and its contacts being treated? (We know it is in the State of Mexico) where was he infected?	No identificado
126	How many people come to Mexico from affected countries?	No identificado
127	Is Mexico in a situation of high alert?	Uno TV
128	Are you aware of PAHO officials who visit Mexico to verify readiness?	Uno TV
129	When would Mexico be ready to be verified by the WHO?	Uno TV
130	Know your opinion on the protection of the new food labeling?	Laboratorio de Periodismo
131	Is the case of coronavirus in the southern medical center true?	TV Azteca
132	Yesterday at the meeting with governors, the states were asked for a list of needs. Could you tell us what the national scenario is?	Revista Proceso
133	What would Guanajuato need?	Revista Proceso
134	Regarding the consolidated purchase, how would it operate? Would the states do it?	Revista Proceso
135	Where does the infected person work?	Excélsior
136	What will be the priority of the national council of health?	Canal once
137	What report do you have at the time of measles cases?	Notimex TV
138	Could you tell us more about the 7th confirmed case	La Jornada
139	How do you define where to buy supplies?	La Jornada
140	Hospitals and the health care system will operate normally for the care of the population?	La Jornada
141	What about public spaces, beaches, for example, cruise ships? Are they still the same?	La Jornada
142	What information do you have about the patient who was isolated from the Pemex hospital and was discharged today?	Vanguardia Veracruz
143	What is the next stage for the cases that have been diagnosed until today?	Canal once
144	What is the situation of the critical case?	Canal once

145	What happens if a person becomes infected with the 2 strains that exist so far in China?	Diario de Nuevo Laredo
146	On Friday, the summit begins in Papantla, and they are worried because foreigners are coming from Europe and Asia. Will there be a protocol?	Vanguardia Veracruz
147	If a person has symptoms and is he going to be in breach?	Vanguardia Veracruz
148	The Mexican strategy that does not limit the entry of foreigners is efficient. Can you expand that?	Medicina digital
149	SARS cov 2 and coronavirus is the same?	Grupo fórmula
150	Why are we not dispersed in this phase? How to explain to people that we must not lower our arms in this phase?	Grupo fórmula
151	About ASA flight 666, how many people got off from Rome?	Milenio
152	What protocols were followed?	Milenio
153	Is it true that there were at least 2 positive people on the flight?	Milenio
154	What happens that they are asking you for a medical certificate to go to countries like Japan? Is there a lack of coordination?	Milenio
155	How many tests are they distributing to the whole country? 200?	Milenio
156	International health did not inform you of suspicious cases on the plane?	Milenio
157	How can we understand that in the United States there is dispersion and in Mexico it seems that we are not as if we were an island?	La Jornada
158	The United States embassy issued an alert to its travelers as if they knew something that we did not	La Jornada
159	Why is Honduras suspending flights from Mexico?	La Jornada
160	A photo is circulating of an airport worker who was detected with coronavirus. Is it real? What are they doing?	Reforma
161	If you are the only ones, who will inform us of what happens with the flight from Italy, for example?	Reforma
162	Right now you don't know if the cases of these flights are suspicious?	Reforma
163	If they meet the requirements, are they tested?	Reforma
164	The president says that there is no makeup of figures, yet your colleagues say there is little evidence. How many tests are being done?	Milenio
165	Why don't we apply tests like in South Korea?	Milenio
166	What about ambassadors and other Mexicans who work abroad? Are they sick?	Milenio
167	How much of the 40 billion public funds would be taken on finance, or would there be another budget?	Milenio
168	What about the purchases that Hacienda announced?	Milenio
169	The WHO has called for the promotion of measures for the production of mouth covers or other materials. What is going to be done in this regard for Mexico and to help other countries?	Radio Trece
170	How are we protecting our doctors and health personnel, with what material resources?	Radio Trece
171	We have information that Trump closed trips to Europe for 30 days. At what point would Mexico take measures like this? Can you be very specific on this?	Forbes Mexico
172	At airports will there be restrictions?	Forbes Mexico

173	Nuevo León confirmed a case and I did not see it in the statistics. What is it?	Reforma
174	The tests that Mexico is carrying out are the ones that it has to carry out, how many are there?	Reforma
175	Can you tell me about the sentinel model?	Reforma
176	How is the market study for the strategic reserve going?	Reforma
177	On the panic that the UN declaration has created, how can it affect the saturation of hospitals?	Reforma
178	The United States does cancel flights from Europe, could they connect through Mexico and could that affect Mexico?	Reforma
179	Gatell said that in phase 1 the economy would not be affected but internationally there is already an impact, can you give me an example of how it would be affected?	Reforma
180	Could you inform us about the risk of recurrence of the virus in recovered patients?	Periódico digital Bajo Palabra
181	After the new measures, in what situation is the San Marco fair, is it suspended?	Metropolitano Aguascalientes
182	We already understand what scenario we are in, but how do you tell people that the WHO elevated this to a pandemic?	Uno TV
183	Given what our neighbor does, could Mexico restrict flights to Italy, Spain, or Europe?	Uno TV
184	For Dr. Ricardo about the healthy distance in scenario 1 like Vive Latino, I do not know if the virus can be spread, I who have lived those events, everyone sweats, so the sweat of one person with another .. Isn't that a problem?	911 noticias (Baja California)
185	What are the protocols for mild symptomatology that is sent home, that is, without medication? or what kind of drugs are they? And if it is being monitored, how is it monitored?	911 noticias (Baja California)
186	when symptoms are severe or acute, is it necessary to go to a hospital? What is the treatment? how long or what does the protocol depend on?	911 noticias (Baja California)
187	The case of Querétaro, in the table there is only one case but I understand that it has already been reported that there is another case in Querétaro	Reforma
188	There are suggestions from UNAM academics that the government should already implement actions for phase 2 of community dissemination. Are they considering doing so? And I ask because both at the Tecnológico de Monterrey and at UNAM, they are already planning to cancel classes, massive events, starting next week	Reforma
189	The two cases that you are studying, could we already speak of community transmission? Autochthonous?	Reforma
190	So what is the rate ... it said that Japan has 66 per million, Mexico?	No identificado
191	are then applied the measures of phase 2, without being in phase 2?	Canal once
192	In light of what the president of the United States announced about a national emergency, what characteristics would have to occur here in this country for a national emergency to be decreed?	Canal once
193	What is the reporting method that private hospitals are using?	Canal once

194	The table shows the case of Querétaro .. one of the cases is the mother .. without a travel history, would it be the first case of community contagion?	Canal once
195	The WHO asks to adopt a comprehensive approach, quarantine, distancing, to do it all. Is Mexico complying with that approach?	Uno TV
196	doubts about those who have already been discharged specifically, how were these people treated or cured?	Uno TV
197	is there a scenario in which the support of the military is anticipated? like DN3	Uno TV
198	Do you have a record of confirmed cases in the ABC hospital of the observatory? there are talk of 9 people positive for COVID	Uno TV
199	how many confirmed cases are there in other private hospitals?	Reforma
200	Although they are saying that they are going to announce the measures in the coming days, of the measures that we have in scenario 2, which ones can they be implemented? school filters? cheers, Hugs?	Reforma
201	There are private hospitals that are already enabled, will Cofepris certifications be given?	Excélsior
202	The question of telemedicine, as are many patients ... can the subject of telemedicine come in?	No identificado
203	In other countries, there are already reports in scientific journals, nationalities document the course of the disease. Is there any report of this in the Mexican profile? in the Mexican genome, how is this virus working and if this can be transmitted to a big data	No identificado
204	one can go to a private hospital and pay for a test?	No identificado
205	The issue of private hospitals seems to me to be very serious ... because today ABC confirmed 9 cases .... (Gattell interrupts, scolds him, and then responds) Dr. Francisco Moreno has already confirmed in interviews 9 cases only in that hospital .. so what are the protocols? Are you informing INDRE? Why does a private hospital say yes, I do have cases of covid19 and you report them as suspects? It is clear to me that you are the authority, but why is this information vacuum occurring?	No identificado
206	... the difference is more than double, isn't it being minimized?	No identificado
207	On Thursday, yesterday and a moment ago, the measures of healthy distance in mass events were reiterated, although public transport is not a mass event, what measures will be taken?	Periódico digital Bajo Palabra
208	if a person is not close to the capital or the clinic where the tests are done, what should they do to be tested or confirm whether or not they have the virus?	Periódico digital Bajo Palabra
209	are there already recommendations from the Helath Secretary to the Secretary of Wellbeing? to the national coordination of scholarships .. for the face-to-face deliveries of scholarships	Victor blogs
210	We are in the middle of a census, what is the recommendation in this regard?	Plenilunia
211	Regarding the measures, would they have to be repeated again? or will it only be once according to the tipping point	Plenilunia

212	What would the conditions to end the pandemic be? We have talked about the increase but when would this end?	Plenilunia
213	Could you tell us who is in this team of specialists? Could you tell us names and something about their profiles?	No identificado
214	to which hospitals would the seriously ill be referred?	No identificado
215	and how are these hospitals preparing?	No identificado
216	What measures are going to be taken at Vive Latino?	Milenio
217	Are the 3000 intensive care beds enough?	Milenio
218	Regarding inputs, they said that market studies would be carried out to identify the costs. If they already have it, what will the cost be? the investment? Where are these supplies going to be brought from?	Milenio
219	It was announced that classes would be suspended, what will happen to the children's stays?	Milenio
220	about the suspension of massive events, the representation 177 of the passion of Christ and I understand that there are no intentions to cancel, despite the fact that around two million people gather there, what exactly are you going to do in Iztapalapa?	Milenio
221	in the case of the subway, what are the controls?	Milenio
222	about pregnant women, from what stage do they have to take care of? what about the product? do you have any permission?	Milenio
223	how much do these thermal cameras cost?	Reforma
224	in how many airports are there? what capacity do the airports have? or why do they intend to buy more?	Reforma
225	50% of the 41 confirmed cases (presented on a slide) ... were between 30 and 50 years old and the majority are in Mexico City, could you give us an explanation about it?	Reforma
226	why the call to "not vacations"?	Reforma
227	Of the cases that occurred today, were any of them community contagion? or are they still imported?	Glucosa Atómica
228	Regarding the measles cases that are appearing in Mexico City, what measures are being taken?	Glucosa Atómica
229	the suspension of classes they announced, what about medical schools? about them also applies?	Glucosa Atómica
230		Animal político
231	In Animal Politico we open a site to verify the information and to receive questions; among the most recurrent are those that I am going to ask you, and one is related to the tests ... if there is not enough evidence, how do you know if you are doing what? More pertinent, the question is, is the decision not to do further testing still feasible or relevant? or what is the explanation for not doing more tests at this time of the pandemic ..	Animal político
232	When will private hospitals be able to carry out covid tests and validate them, without the need for them to go through INDRE?	Animal político
233	yesterday it was announced that from the 23rd the essential activities will be suspended .. what are these activities? is it a recommendation or is it mandatory?	Medicina digital
234	what about Campeche? why are you not reporting data to the system? Are there problems with Governor Alito?	Medicina digital
235	Today at 1 in the afternoon they released the report of positive cases, it is striking that Mexico City has 17 positive cases ... of course it is not my intention to ask you about this	Heraldo de México

	situation because that corresponds to the Secretary of Health from Mexico City, thank you for introducing it to us, the source did not know it ... I hope the secretary will attend to us and tell us what will happen to those 17 cases, but if you have any comments ...	
236	Where are the severe cases of coronavirus located? at least public or private hospital?	Heraldo de México
237	Dr. Cortez spoke of not leaving the children in charge with the grandparents. Could you give us details of that recommendation?	Heraldo de México
238	Has the estimate changed, what have they done with the resources needed to address the situation in the country? You had said a few days ago, in a scenario different from today, that an additional 20 million more would be needed at most if we entered phase 3. Has this perspective changed? Need more money? Even today, Mexico City said about 100 million pesos.	Milenio
239	If Donald Trump closes all the consulates, what is going to be done there?	Milenio
240	A publication came out that Mexico occupies the contagion ranking of 182 cases, this was published in NYT, does this refer to the cases that live there? or if you can explain to us ... this ranking in which Mexico occupies 5th place	Milenio
241	In the same investigation, it is published that there may be up to 5 or 10 cases that go unnoticed for each positive case. If you can clarify	Milenio
242	the state of health of the sick person ...	Milenio
243	In what stage can we consider that we are in scenario 2?	La Jornada
244	Can you explain why the rapid tests worked for Korea?	La Jornada
245	In the case of Mexico, how awill weknow about community transmission if only people who have traveled or have had contact are tested?	La Jornada
246	What is the mechanism that older adults living in nursing homes or nursing homes must follow?	Periódico digital Bajo Palabra
247	About the development that is being carried out in other countries, in this regard, you know that in Canada and the United Kingdom, they have freed up resources for research on COVID, for example, genomic epidemiology, development of new proteins, etc. Has this contingency aroused interest in the government for development against the coronavirus? as well as in influenza for which you already have a vaccine	Periódico digital Bajo Palabra
248	Is there a budget for long-term coronavirus care?	911 noticias (Baja California)
249	In the coordination scheme of federal agencies, would conago enter there? (national conference of governors)	911 noticias (Baja California)
250	in scenario 2, would we be entering phase 2 this week?	911 noticias (Baja California)
251	the Pacific zone continues to appear as yellow, but this figure has increased ... it is a part where it has not evolved ... they only continue to be suspicious and suspicious ... why is it that they only continue to be in a state of suspects?	Canal once
252	How is the report going, the count of the states that were going to report what they were going to occupy to attend to. What are they most reporting that they need?	Canal once
253	TThe schools will resume classes tomorrow; they spoke with the SEP about how this week will be. Will the	Canal once

	classrooms be sanitized? will there be gel at the entrance? and what measures can parents take to join this strategy?	
254	How many of these cases are reported serious and where are they, public or private?	MVS
255	Is there a possibility that the suspension of classes is advanced? especially since some states have been signaling that they are going to suspend starting tomorrow	MVS
256	They are reporting that due to the closure of borders, some countries in South America and Central America are reporting, people have been left stranded at the airport. What is going to happen to them?	MVS
257	What do you reply to opposition parties that have been requesting your resignation due to the statements you gave this morning?	Excélsior
258	When is the CSS going to meet to adopt measures for the whole country? And thus avoiding chaos, because right now, the universities, the private sector, the governors, and now even the church is taking measures that they consider more pertinent without being a guiding axis or they do not want to do so as not to restrict the president's tours?	Excélsior
259	Do you have a meeting, do you have an appointment with the CSG or will it be until they decree a health emergency?	Excélsior
260	there is a lot of concern about disabilities because workers are coming back with flu, and they are not giving them disabilities	Excélsior
261	How will the corpses of those who die from coronavirus be treated?	Grupo Imagen
262	the 800 phone is saturated ...	Grupo Imagen
263	If from Friday to Saturday, we get from 20 to 82 cases. On that scale, when would we enter phase 2? already with national cases ...	Medicina digital
264	Thank you for the table of inputs that will be required; the question is, in the 2020 budget, were not part of those inputs already contemplated? ... the question is not free, today in the INER there was a movement of The workers who even removed the director of the Institute because they have ways of working that one would not imagine there, right? There are no protocols; they complain that they are working without gloves, they do not have goggles, gowns ... we are talking about something more serious .. it is a hospital that treats infectious diseases, so it is not possible that our workers, health workers, are working in these conditions .. and that plans are made here everything looks very nice, and the truth that we want to see implemented. Thank you very much	Contraréplica
265	this list of supplies for what time would we be thinking?	Contraréplica
266	The health sector still does not need the 25 million pesos that the Chamber of Deputies has offered?	Eje Central
267	I know you have said it on other occasions but I would like it to be more precise, what is the contingency plan that you are doing for when the time comes to implement phase 2?	Eje Central
268	How much is the budget for this phase?	Eje Central
269	Is it going to compensate in any way the families that live day to day like in Peru? in addition to the 81 confirmed patients that there are so far	Eje Central

270	How many people who have been in contact with them are still in quarantine?	Eje Central
271	What about tests to identify symptomatic carriers? ... based on antibodies and detect genetic material ... how would they be evaluating them?	MVS
272	This family outbreak that you mentioned corresponds to these 9 cases that were the contacts? if you could clarify that for me	MVS
273	part of the demands today at the INER was that they had not even been informed of a protocol on how to treat a person in a suspected case and subsequently a person who had been confirmed, specifically what protocol? Which document? Who is informing medical personnel how to treat these cases and how to protect themselves from getting infected?	Grupo Imagen
274	Of the 93 confirmed cases, what number are serious?	Grupo Imagen
275	In scenario 3, where you are considering 250,000 potential patients, what number of ventilators are needed? what number of beds? do you already have everything? or will we wait for stage 3?	Grupo Imagen
276	Pregnant women are supposed to be in the risk group. If a pregnant woman has the symptoms of coronavirus, does it affect the baby?	Grupo Imagen
277	can you breastfeed the baby? What are the care that should be taken?	Mañanews de La Octava
278	do you plan to start doing open data? base csv, etc.	Mañanews de La Octava
279	they have contemplated at some point doing mass testing, which the WHO suggested ...	Mañanews de La Octava
280	In social networks there has been talk of the quarantine ... are we in quarantine or not?	Mañanews de La Octava
281	What should tourist destinations do?	Milenio
282	Is this scenario a conservative scenario? because in the first scenarios they presented to us they told us about higher estimates	Milenio
283	Scientific evidence indicates that for every person who is identified, there are 10 who are not diagnosed and of the people who are apparently cured have lung lesions ... those are things that have been identified in China ... if you can tell us. .	Milenio
284	If you can tell us about the meeting or videoconference that the secretaries of the health of Mexico, the United States, and the Minister of Canada had	No identificado
285	In any hypothetical case, if an ambulance can deny service to a patient with data of respiratory distress, suspected of coronavirus?	No identificado
286	Do the ambulances in Mexico have the capacity to transport infectious and contagious patients?	Plenilunia
287	Can you tell us something about the health council meeting?	Plenilunia
288	with regard to materials in other indigenous languages, Nahuatl, Mayan, which has also been a requirement at different political levels and which is also necessary	Plenilunia
289	If the doctor can tell us about the strategies to apply care but to people with chronic diseases, cancer, etc. required by other patients.	Grupo Imagen

290	I would like to know your position if, as the Secretary of Health, you are assuming, on the video that the governor of Jalisco recorded, warning about 400 people who could be infected with covid19 and who could be dispersed ... the video summons the people who traveled to Baja California to report for the study to be carried out and to avoid a massive dispersion of covid19 because there have already been several cases of families who went precisely to enjoy a few days in BC, and that confirmed cases are being presented	Grupo Imagen
291	Today they promised to bring the data about the number of ventilators and intensive care beds that you have already ready if scenario 3 occurs in this contingency ...	Grupo Imagen
292	If you allow me, I know that you had said that it is the Secretary of Health of Mexico City who is in charge of the measles issue, however, today a case has already been detected in the State of Mexico, and I would like to know what do you have as a federal agency	Milenio
293	I am receiving information that I would like you to - I adhere to transparency - inform us about a 42-year-old person hospitalized at the INER, who was infected on March 2 in England, is a chronic diabetic person, we would like you to confirm us if this is real if there is a case like this	Milenio
294	If you can tell us, how many of those who were infected in Bayl, Colorado, is Mexican and they already know the route from where the first contagion was	Milenio
295	If Dr. Ruy can tell us if this virus has already mutated, there is a publication that has already mutated in Spain if you can answer us	Milenio
296	The closure of the borders between the United States and Canada, Donald Trump says that he may make a border closure for health reasons towards Mexico. What would be the burden of expelling the migrants or deporting them? This is being discussed in the CSG?	Milenio
297	Right now it's 7:38 if you can confirm us, with all due respect, if you can confirm us before the end of the conference if the person died? ... I believe that speaking to the INER it is solved ...	Reforma
298	Are infections affecting people located above latitude 22 of the planet? some studies have published it ..	Reforma
299	there is a gap between what the states report and what the SS reports, Nuevo León for example	Reforma
300	how many people are serious?	Reforma
301	Dr. Gatell spoke of 3,000 drugs to be bought	Reforma
302	which hospitals are already being converted?	NPR-Noticiero argentino en Estados Unidos
303	how many tests have you done?	NPR-Noticiero argentino en Estados Unidos
304	still have 9100 tests?	NPR-Noticiero argentino en Estados Unidos
305	reactions or tests?	NPR-Noticiero argentino en Estados Unidos
306	where did they buy them?	NPR-Noticiero argentino en Estados Unidos
307	how do they know to go to phase 2 if there is no massive testing?	La Jornada
308	About yesterday's death there was a lot of confusion and it is still not clear if you could tell us if this person had a history of traveling abroad or not? ... or if he qualified for external cases or qualified as a case of community	La Jornada

	transmission that is not you have registered .. and what is the reason it was not included	
309	have considered modifying the closing time of the report ... because closing it at 1 and giving us a report at 7, there is a very large window that I do not think ... tell me if it is feasible, reliable or convenient in terms of the clarity of this window ...	La Jornada
310	about the tests ... I want to insist ... there is no need to do an intentional search for people to avoid a greater community dispersion and thus anticipate prevention? and to a lesser involvement of people.	Milenio
311	What about the wife or partner who was in contact with the person who died? who was even in contact with the media ... there is no isolation process	Milenio
312	Right now, there are serious protests from the INER and the ISSSTE, and different hospitals complain that there are no supplies. People also complain that they pay for supplies ... if a unit denies care ... what responsibility could fall?	Milenio
313	The United States and Canada have already declared a state of emergency, you have told us that this is not an emergency, have you not considered changing your strategy?	Plenilunia
314	Is the CSG going to be a permanent measure or was it closed? would they mark the line of what follows?	Plenilunia
315	There is confusion about the laboratories, whether or not they can do the tests ... if you can clarify for us ...	Plenilunia
316	I believe that I have not done my job well or we have not done our job well because a judge is asking for more information about the preventive measures and actions and they want more information and presenting more actions, also your comment on the matter ...	Grupo Imagen
317	first a precision for Dr. Alomía, when you that the person who died yesterday was not at festivals, do you mean Vive Latino? because we have the report -you tell me if it is false- that this person attended a festival at the Palacio de los Deportes on March 3, and then there was some other type of contagion ... because I understand that he presented the symptoms on the 9th, but the festival was the 3	Grupo Imagen
318	If the CGS met, does it mean that we are nowhere from entering phase 2? This could be announced between today, tomorrow or the weekend?	Uno TV
319	What would be the response for this judge who is demanding that in 24 hours report on what measures have been taken with the covid19?	Uno TV
320	what other things did you agree on at the CSG?	Uno TV
321	The president spoke about the DN3 plan, and I had asked 3 weeks ago if they would use it. What is this plan about?	Ni1+
322	A trend is being released with rapid tests to detect the coronavirus, they are being offered up to 1500 pesos per piece, it is a very simple one to check glucose, or HIV, it has the same system, what does the federal government know in this moment? What is being done to stop this trend? Are they reliable?	Ni1+
323	The federal government has asked society not to make panic purchases, not to spread fake news. It has also presumed the synchronization it has with the different	Glucosa Atómica

	levels of government. In the states, what happens when the government sows psychosis and terrorism? Maybe they have the data, yesterday the mayor of Sinaloa, Manuel Chartman Moreno, along with his entire cabinet, the health sector, social communication, and the police elements, came out with white space suits, face masks, to alert neighborhood by neighborhood .. panic today is sown by the mayor, the SS will issue a fine for violating the procedure they have recommended?	
324	Do you have any report of a lack of supplies in the hospitals?	Glucosa Atómica
325	It was confirmed that there is a positive patient where the isolation was not correct, doctors and nurses had contact with him, how are they going to protect the health sector? and how are they going to isolate positive cases?	Glucosa Atómica
326	The SS of the Cdmx issued a statement saying that undergraduate interns are going to suspend activities. On Sunday Dr. López-Gatell announced that they would not enter into this suspension, what can you tell us about it?	Milenio
327	How much would care cost for patients who are already seriously ill?	Milenio
328	What is the alternative that is regularly attended in nutrition, for example in Baja California, who do not have respiratory diseases but who need medical attention ..	911 noticias (Baja California)
329	Several acts of vandalism, robbery and everything else related to scenario 1 have been reported, I don't know what could happen in scenario 2 or 3, is there any protocol or measures that have been taken as a security committee?	Canal once
330	Can you remind us of what the hospital reconversion of these 4 hospitals consists of? what will they have?	Canal once
331	This week, what will happen to the appointments already scheduled at the IMSS, the ISSSTE?	Uno TV
332	In the morning, at this conference, are you going to respect this healthy distance you are advising?	Uno TV
333	when could you expect the peak, the crisis, the thousands? and in what other month would the decline happen?	El Universal
334	has insabi consolidated a purchase? how many inputs were purchased?	Milenio
335	An agreement is circulating with the public sector for the suspension of work from March 23 until April 19 to know if this is real.	La Jornada
336	He touched on a point that may be a bit contradictory. What will happen to the personnel who work in the federal government? Is there an agreement for these people to work from home?	La Jornada
337	We had been announced that the implementation of the sentinel model is coming, could you explain a little more?	La Jornada
338	about the elderly, how should your routine be? How to keep a healthy distance at home?	No identificado
339	Could you confirm if there are confirmed or suspected cases in the armed forces?	Multimedios Televisión
340	about SEDENA, is special equipment already considered?	Multimedios Televisión
341	The following week, we enter with the healthy distance measures, many people will not be able to enter because they work in informal commerce, what recommendations	Milenio

	would there be? Do you have any estimated support for these people?	
342	What will happen to the medical supplies? free import is allowed?	Milenio
343	Is there going to be the intention to go with the ambulances to look for the patients who have symptoms in an intentional way?	Milenio
344	PAHO increased the risk of people falling into critical risk from 5% to 7%, due to diabetes and obesity.	Milenio
345	Regarding the case of INER, of the 41-year-old, is it known where he acquired the contagion?	Revista Zócalo
346	Taking advantage of the Dr. Do you have recent information about new fake news about the coronavirus and who would be generating them?	Revista Zócalo
347	yesterday there were 49 cases and today there are 65 more, what trend do you hope will prevail in relation to the stagnation and growth of cases	Excélsior
348	Today the journey of healthy distance begins, but a few meters away we still see many people, it seems that this distancing has been taken as a vacation, what would be your opinion?	Excélsior
349	Regarding the first case that died, it was striking that the wife said that she was not tested and also was not in isolation, was she tested? because it was not quarantined?	Excélsior
350	AMLO said that the army will have 10 hospitals under its charge, what are they? Will they be served by the military or civilians?	Grupo Imagen
351	there is a document by workers of the attorney general of the republic, where they ask the prosecutor to try to support with some measures to help ..Is there any indication that there will be groups that can leave alternating in the population in the next few days? To what extent is the federal government contemplated to continue working?	Grupo Imagen
352	Have you detected cases within the armed forces or security groups? or in the state police?	Grupo Imagen
353	people lose their sense of smell and taste? or are the symptoms exclusively those that we have been told?	Plenilunia
354	What are the agreements that are being carried out with the laboratories to make the process more transparent?	Plenilunia
355	what is the position of the SS regarding the tests approved by the FDA	Plenilunia
356	What would be the strategy suggested to the States for homeless people?	Multimedios
357	Dr. López-Gatell commented a couple of conferences ago that the quarantine could not be brought forward due to the economic impact it would have on the country, that is, it would be more worthwhile to wait in a slightly stronger scenario, then pause the economic activity, how long would this quarantine last? this economic break, a week, two, a month?	Multimedios
358	There is a lot of confusion between the percentages of infected. We know that an average is 80% simple recovery, 15% with hospitalization, and 4, 5, or 7%, in our case, intensive therapy. Many people are confused because they think that this 100% is of the population. They handle	SPR - Canal 14

	numbers based on the country's population to talk about people in intensive care, but we would be talking about millions. Are we talking about those figures? that is, the general population is not a population of infected	
359	He already spoke a little about the laboratories .... at what point do you have a date to have access to those laboratories that are certified?	SPR - Canal 14
360	Due to the shortage of antibacterial gel, home-made products are already being sold, even outside the subway, face masks out here. How do you plan to control this sale?	Canal once
361	yesterday the federal government informed that it would hand over control of 10 hospitals to the armed forces. I don't know if they are in a position to give us the names?	Multimedios Televisión
362	Regarding all these recommendations, from a healthy distance, etc. What is your position ... do you consider that it is a bit incongruous with these measures of healthy distance and perhaps that is why they are not being followed by people? with the example set by the president who has already caused much criticism at the international level	Multimedios Televisión
363	They recommend for people with mild symptoms that they should stay at home ... however, these people, who at a certain moment would require their disabilities, present them at their jobs ... how are they going to do it? if in addition they are not going to carry out the tests?	Reforma
364	We already entered the second phase, or when would we enter the second phase?	Multimedios
365	Many people use scenario 1 in Italy comparing it with the current scenario in Mexico, and if we consider that in Italy more than 4000 people have died, even more than in China, since this figure makes people very afraid, the factor that many in Italy had diabetes, cardiovascular problems, etc. This can be interpreted as for people to compare the scenario of Italy? what is the interpretation? Why is it not compared to Mexico?	Revista Zócalo
366	From the projections you told us, you tell us that 42% are already in America ... what would your projection be for the following weeks, if within a few weeks Europe will cease to be the main focus and perhaps America will become a new focus of expansion and growth	Revista Zócalo
367	How many serious cases have you currently registered? because unfortunately the serious ones are headed, some, towards death ...	Revista Zócalo
368	Yesterday they told us that they increased 60 cases, but from yesterday to today they only increased 16?	Revista Zócalo
369	despite Claudia Sheinbaum's recommendation, some restaurants are still open .. your exhortation please	Canal once
370	In this new material on counts, there are 5 of the 377 .. what number would we have to reach to declare phase 2?	Canal once
371	the 01800 phone is already reestablished?	Mañanews de La Octava
372	on the transition of the phases ... some local governments use the term "curfew" ... are these provisions going to be made at the federal level?	Mañanews de La Octava
373	There is a forecast from Mtro Jorge Galván that projects that at the maximum peak we could use 86,000 beds. Do we have that capacity?	Mañanews de La Octava

374	In the chat groups of the health personnel he feels very abandoned ... I know the screenshots from a good source ... I know that they do not want to abandon them .. but what would it be ..	Grupo Imagen
375	Today, the WHO director said that social distancing measures are insufficient. In fact, to win, you have to attack with well-directed tactics, test suspected cases, isolate and quarantine close contacts. What is going to do Mexico in the face of the WHO declaration?	Grupo Imagen
376	How do we explain to the population that we are entering phase 2? what would be those indicators that would make you indicate that we are in phase 2	Diario 24h
377	Why have several states of the Republic, including Mexico City and the State of Mexico, have decreed their own hygiene and sanitation measures, would it not be better for the federal government to take a general measure and for the other states to converge on the same?	Diario 24h
378	You put the cases of Mexico in, I do not remember which slide. Do you already have a record of some cases that are already relieved? Is this data available?	Diario 24h
379	Is it feasible for the Mexican government to continue applying the tests? The United States is no longer applying them.	Medicina digital
380	Why isn't the sentinel data released to us? The sentinel system has been in place since 1994, it has an official Mexican standard, we have monitoring units, the objectives are clearly established, which are to search for the virus, so ... why don't they release the data? and I tell you this because - don't let me lie - epidemiology is not an exact science ...	Medicina digital
381	have covid19 cases been detected in this system?	Grupo ACIR
382	the health status of the 3 babies who were diagnosed, one in Jalisco who came from Spain	Grupo ACIR
383	the army is going to take 10 hospitals, what are they?	La Jornada

Phase 2:

1	Until Sunday there were 3 babies and the one from Yucatán is no longer there. What about the data there?	La Jornada
2	What does it tell you about the governors making decisions for their part and that there is even talk of a group of governors who want to make an independent bloc to take their own measures? It's a political question	La Jornada
3	On the well-being doctors to hire, those who have registered, more than 5 thousand if I remember, would not they serve for this call? "What are they thinking?	La Jornada
4	Can you give us the number of death people by age ranges?	La Jornada

5	And if they can specify the origin of the infected, the source of transmission is not specified	Diario 24h
6	You said that Mexico was able to plan the stages. In that sense, the projections change or remain the same	Diario 24h
7	In the graphs you can see how it grew in Spain and Italy. The distancing there is not working?	Diario 24h
8	Is Mexico contemplating doing the rapid tests like South Korea?	Grupo Imagen
9	If only one sample is done, is there not the risk that the numbers are underestimated and people infect others because they do not know they are infected?	Grupo Imagen
10	In the case of rural areas, how is this system going to be applied in these areas to prevent COVID-19?	La Jornada
11	What makes you think that people have not understood? What gives you that perception that they have not understood?	La Jornada
12	Does the closure announced for tomorrow apply to everyone? How is the government going to actually make people stay at home in the private sector?	La Jornada
13	Is there more data on immunity in recovered people?	Milenio
14	Tomorrow how many people would stop being on the street?	Milenio
15	These 13 people who do not know the origin of the contagion, where are they from? what states are they from? And if they have contacts?	Milenio
16	Will the people who will receive this leave or disability be 100% of their salary or 60%? How shall it be done?	Uno TV
17	Starting tomorrow, the federal government will stop. Does that include the AMLO conferences?	Uno TV
18	People who have to go out because of essential work, will they have to wear face masks?	Uno TV
19	What is certain that in COFEPRIS a shipment with supposed medicine for COVID-19 is detained?	Canal once
20	Regarding deaths, that table already includes those of CDMX and Edo Mex, which were the last?	Canal once
21	About the people who are in the streets, what would be the recommendations for their protection?	No identificado
22	As head of the Intelligence Unit, I would like to ask you how the number of infected and deaths is confirmed? The governor of Jalisco speaks of 1500	No identificado

	cases that the authority has not confirmed. If you can delve into the conformation of the figures	
23	They are talking about antiretrovirals and malaria medicines, what can you tell us about that?	No identificado
24	What is the protocol in food handling?	No identificado
25	In Nuevo Laredo it is said that there is an international airport and a flow of daily trailers that pass from 50 states of the USA and other states of Canada without control...	Audiorama
26	What would be the explanation for all those people who question the numbers they present to us, especially on the border where there are few on the Mexican side and not on the other?	Audiorama
27	What do they say on the networks about coming home and taking off your shoes, do they work?	Audiorama
28	There are companies that are laying off people, is there a call from the health sector for these people not to be left helpless?	No identificado
29	Versions about an alleged modification of the health sector regarding the increase in pneumonia compared to 2019 have circulated in networks	Multimedios
30	Difference between typical pneumonia and COVID.19?	Multimedios
31	Are new medical personnel infected?	Eje Central
32	Since they detected the first case of community transmission, have the number of tests increased?	Eje Central
33	If it is no longer necessary to have a foreign travel history, will everyone who goes to the hospital be tested?	911 noticias (Baja California)
34	Yesterday you read a letter of solidarity with the health workers who are demonstrating, however that is not enough for them, what else will be done?	911 noticias (Baja California)
35	Los Cabos as a tourist destination has had an increase in cases. In that sense, is something going to be done for the highly touristy states?	911 noticias (Baja California)
36	About the coexistence they spoke about yesterday, IMSS, ISSSTE, and SS say that they have the medicines, but it is not true, and proof are these prescriptions sent to us by people who do not have to pay for the medicines. I would like to give you the prescriptions and know if they can help those people who take antidepressants, antipsychotics and	Excélsior

	anxiolytics and know if those medications are going to be dispensed.	
37	There is nervousness with the doctors, and they ask me to ask you. If they are going to be given priority to treat them if they get infected?	Excélsior
38	In Spain 10% of health personnel were infected. They also want to know how you are going to reduce the risk? They are very nervous because they see that those who work in the private sector have better suits than those in the public	Excélsior
39	They say a dead patient arrived and was not tested. I want to ask if the autopsy will be done and the procedure to count these infections?	No identificado
40	Until last week there were 3 minors with COVID-19. They have any update of babies under 1 year	No identificado
41	The Mexico Government has a plan for women who go into isolation and cannot contact the baby. Is there a plan?	No identificado
42	Like what plans?	Milenio
43	You have told us that the peak will be seen at the end of April, is it among the plans to extend the health distance?	Milenio
44	Today in the USA the use of blood plasma was authorized as a treatment, could this be used in Mexico?	Milenio
45	So the peak will not be the same or at the same time in all of Mexico?	Notimex TV
46	Why is there so much contrast on the border between the United States and Mexico? This is due to lack of evidence?	SPR - Canal 14
47	What happens to people who do not have social security schemes. Where can they go for medical attention?	Reforma
48	How many disabilities have been granted in IMSS by Covid, by influence, and by SARIs?	Reforma
49	What are the requirements for these people to have access to this disability?	Nación 14
50	You have mentioned that by April 18 we would already be talking about the maximum peak, does the Ministry of Health have an approximate number?	Nación 14
51	How many cases would the Ministry of Health talk about to go from the second to the third phase?	No identificado

52	The method of counting deaths, the cut-off, is made at 1:00 p.m., does it lend itself to mistrust that some cases are included, others are not, and there is some confusion that the federal government says some and the governors others?	No identificado
53	The subject of fans already know if they are going to buy more?	Periódico digital Bajo Palabra
54	It seems that a large number of Mexicans are not respecting the call to stay at home, do you think it is worthwhile to insist on this call or if it is necessary to expand it?	Periódico digital Bajo Palabra
55	I would like to know your opinion regarding the criticism that President AMLO has received by international organizations that call him a hazardous example that threatens the health of Mexicans for not complying with health recommendations and if his call for all of us to abide by the prevention measures also includes the president?	Reforma
56	The governor of Hidalgo tested positive; he was with AMLO in the morning. Is he going to do the test? Or will you take isolation measures?	Reforma
57	But this happened last week, shouldn't he be tested?	Multimedios
58	Could you give us an estimate of how many deaths there will be? tens, thousands or millions? I say to reassure people please	Multimedios
59	But in down-to-earth figures?	No identificado
60	They say deaths from atypical pneumonia have increased. Are COVID-19 deaths mixing?	No identificado
61	Are you sure that the deaths reported as pneumonia are not due to covid? On what would be based the certainty that they are not mixing them?	No identificado
62	He reiterates: Are you certain that they are not mixing them?	AVA Noticias - Veracruz
63	Will the back to school date be moved to April 20?	AVA Noticias - Veracruz
64	Is the call for doctors still open? Will other medical specialists be able to fill the templates that are not filled?	AVA Noticias - Veracruz
65	Foreign Minister: How is the repatriation of Mexicans going? the one in Peru?	La Jornada
66	Why do you reiterate this urgent call with such clarity? What do you see?	La Jornada

67	Chancellor: how is the management of supplies abroad?	La Jornada
68	Will there be protocols for funerals?	Milenio
69	More severe measures for the population to stay at home?	Milenio
70	Are you thinking of repatriating Mexicans from other parts of the world?	Milenio
71	How much is being invested and how many protocols are there in Mexico for the cure of COVID-19?	La Jornada
72	Are there ways to identify the sources of infection, to control new outbreaks where the origin is unknown?	La Jornada
73	If there has been a change in the population from yesterday to today on whether to stay at home?	La Jornada
74	Why in Mexico is public force not used to guarantee that people stay at home? When will you do it? Is there a need to take risks?	SPR - Canal 14
75	How will the management of the beds be in conjunction with the Navy or Defense?	SPR - Canal 14
76	Where are they going to build the new hospitals that AMLO spoke about?	Multimedios Televisión
77	Gatell said that AMLO will not be tested because he has contact with the Governor of Hidalgo. AMLO shouldn't isolate himself and be considered suspicious?	Multimedios Televisión
78	It has been reported that plasma will be used for treatment, do you have info about it?	Multimedios Televisión
79	Are there still 5 confirmed IMSS health workers or are there more? Is it negligence?	Milenio
80	Who are the 20 people who died? Where are they from? Because Quintana Roo notified some dead and we don't know if they are here?	Milenio
81	Where are the 132 people who have no travel history?	Milenio
82	Why not take more restrictive measures if Austria - with a history of fascism - is doing so?	Reforma
83	At what point do we enter phase 3? What conditions will there be?	Reforma
84	What is your opinion on Yucatán's decision to tighten the measures?	Excélsior
85	A document is circulating that says that the government is going to take control of transport, food and companies, I want to know your opinion on this	Excélsior

86	How much does the conversion of hospitals cost? What is required and what do they have? hospitals, beds, supplies ...	A tiempo TV
87	How long would it take to convert the hospitals?	A tiempo TV
88	If hospitals overflow, what will happen to those who do not have a bed?	A tiempo TV
89	Are they not expected to ever overflow?	Revista Zócalo
90	Do you recommend any medicine for those who are sick at home?	Revista Zócalo
91	What is going to happen to the patients with other diseases that are in the hospitals to be reconverted?	Reforma
92	What will be the damage if the measures are not taken?	Reforma
93	How do the private and public sectors support the new measures?	Reforma
94	How many infected / recovered health personnel are there?	Milenio
95	What would be the consequence of not complying with the measures?	Milenio
96	How can we protect the most vulnerable (heart disease, hypertension)?	Milenio
97	Are your operations also for the safety of people?	El Sol de México
98	Regarding how companies could act, can they lower wages after today's measures?	Notimex TV
99	Will there be sanctions in the country as there are already in Yucatan?	Notimex TV
100	The protocol of the people who die? is it forced to cremate?	Canal once
101	In this period, are administrative sanctions contemplated?	Canal once
102	What is expected of Mexico abroad in this national emergency? Help from other countries?	Diario Locutor
103	How much will poverty increase in Mexico?	Diario Locutor
104	What would be the loss of the companies with these measures?	La Jornada
105	Ask for an update on the recovered people; it remains 4%, gender, a test was applied to verify if they are still infected, how, what is the number of days, 14 or 28 days in isolation.	La Jornada
106	Of the calls to Social Security, how many are about suspicious patients? are there statistics?	La Jornada

107	How big does the curve have to be on April 30 to predict the next measurements?	Nación 14
108	Essential activities and no ... is the mining activity going to stop?	Nación 14
109	Global Respirator Deficit ... Why not look Nationwide? there are groups, even in Sonora, that hope to develop patents	Notimex TV
110	Now that it is a declaration of emergency .. Will there be an order to close beaches?	Notimex TV
111	will there be follow-up to the order that people can be absent with pay?	Notimex TV
112	What message do you give to the population so that they do not fall into panic buying?	911 noticias (Baja California)
113	This type of intervention is a type of measure that is being taken in Mexico City, but what would be the other intervention panorama? How far could we go? What would be a borderline intervention?	911 noticias (Baja California)
114	I gave some prescriptions to Dr. Borja from the IMSS, who told me that he would contact the person because the medicine has not been given to them, especially due to the lack of resources and it was last week and I have not heard from it yet ... and prescriptions keep coming to us, there are no drugs, there is no efficiency, and they are negative by any health system, except the private ones obviously, but the idea is that it is said that there is health care but there is no ... that's why I was the interlocutor and he has not answered yet, I hoped he was here, but he is not	Canal once
115	Faced with the pandemic, defective products have been bought, the government has also announced that it is going to buy products from this country, how does the Mexican government guarantee that these products are effective?	Canal once
116	On the subject of reconversion of hospitals, here it was announced that they were analyzing the military of Tematla the next COVID hospital, and dir. ISSTE said that a hospital in Cuernavaca, Morelos "El Calero" was also being analyzed for COVID, is that true? How long would it be? Would you join the network of 6 hospitals that Dr. Reyes Terán had announced?	Pulso saludable

117	I read in a Harvard publication that said that the virus can be biphasic and that the hormonal aspect, testosterone, androgens, in which most deaths occur in men, what would be your opinion about it?	Pulso saludable
118	There are several states that already have the same applications, they are thinking of converging all of them and this could help them in the future to establish more precise statistics, I do not mean that they are not doing their job well but that this can facilitate those cases that found in distant places and that they have the possibility of a cell phone	Diario 24h
119	How are the purchases of supplies, both for protection such as medical ventilators and others, if they found the suppliers had said that there were suppliers that are analyzing them a lot or we were late to buy them, on the one hand, to buy supplies	Diario 24h
120	what will be the responsibility of private hospitals? The health sector is helping with private hospitals ... because yesterday a day-old baby was transferred from a private hospital to a covid hospital	Milenio
121	They told us that there were 39 health workers workers who were infected, we would like you to tell us right now how many are currently in Monclova, how many are also specifically, they told us about three cases that had died	Milenio
122	the baby if it was a positive case of COVID	Diario de Nuevo Laredo
123	civil society and they gave me a letter to send it ...	Diario de Nuevo Laredo
124	their experience ands the health authority available to establish links with these citizen organizations and take advantage of both their experience and their presence in the field and provide them with training, where necessary, with inputs so that they can help to contain this activity?	La Jornada
125	Have any differences between the strain that is circulating in Mexico and the one that arrived from China been found at this point in the examinations that the laboratory tests?	La Jornada
126	How many tests are done per day in the country at INDRE in particular?	La Jornada
127	About the 50,000 fans, if you can tell us, what is the process is already being distributed	La Jornada

128	To Dr. Schwartz, how are you doing with the Health for Wellness program? because it is a program that started days ago ... how many people have already hired?	Grupo fórmula
129	Regarding deaths, if you could tell me, they were all hospitalized, some died at home and how quickly the patients have worsened; today 13 people died, yesterday were 8, how's that?	Grupo fórmula
130	What call would you make to the government of the capital to the state governments to avoid this type of demonstration that undoubtedly put the population at risk	Grupo fórmula
131	I understand that strains were purchased, SARS-COV2 strains will be purchased	Grupo fórmula
132	what do you answer to the governor of Jalisco? which mentions that you could be betrayed by not doing massive tests	Telemundo
133	Regarding the population most vulnerable to the epidemic, the first is given the shortage of drinking water in the country, what measures does the health council have in place to ensure supply or, failing that, what suggestion can be made to those people who do not have access either water nor how to access antibacterial gel	Telemundo
134	Which protocols are planned or which ones suggest they be adopted in places where there is a vulnerable population in places with a high population concentration such as popular neighborhoods, shelters for homeless people, migratory stays	AVA Noticias - Veracruz
135	they talk about various medications and they mentioned a method called transferon, I don't know if they know it, if they can give us a little of it as a possible treatment	AVA Noticias - Veracruz
136	question addressed to the INSABI specialist, if they are seeking to modify their protocols so that experienced people can join the call and care for possible patients	Grupo fórmula
137	on the use of the mask while Asia says China Japan South Korea assure that this has been a central factor in reducing the pandemic in the West, it seems that we do not agree what is there about this	Grupo fórmula

138	When will we be able to have a vaccine and a treatment is being talked about these days about a treatment based on plasma-derived from recovered patients, this is true or false doctor	Grupo fórmula
139	the last one that has to do with the truth or the falsity of the information: what we can add to our work so that people do not fall into disinformation	Audiorama
140	what happens with patients who are already discharged if they have a special follow-up, if they have to take certain care or they can simply go out on the street	Audiorama
141	What happens if a colleague with symptoms is detected, the government supports him and follows him up, thinking that the majority of fellow journalists do not have insurance ... and if another way of holding these conferences was considered?	Audiorama
142	Regarding the call that they made yesterday and they told us that there was no limit for these people, I want to assume that it also implies hiring retirees in the health sector, but will they have an age range? so that they are in the range of people with danger of contagion	Crónica
143	In this reflection that you make of these 15 days on the measures taken, you conclude that you are satisfied with what is how society acted	Crónica
144	This is a recommendation now that the rains are coming	Milenio
145	How much do these specialists earn on average and if, in addition to these two additional points for the exam, it is expected as an incentive to increase the salary during these six months that they will be hired	Milenio
146	60% of the deceased ... are not exactly older adults as expected, and if this would force to rethink the situation somehow	Milenio
147	the main cause of comorbidity I have seen has been hypertension, is there a drug that could aggravate the situation, was that matter analyzed?	Reforma
148	I want to know what the salaries are in the health secretariat and if they plan to standardize them during the epidemic	Reforma

149	how much would be the salaries for nurses and how many nurses do they plan to hire or are needed for this epidemic	Reforma
150	I want to ask you if young health professionals are deciding to join this call or how they are reacting to it	Reforma
151	what follow-up is given to mild cases that are not tested ...	Reforma
152	To the teacher Zoé Robledo, from the announcement he made today, was there no more infected in Monclova?	Reforma
153	Of those isolated in Piedras Negras, none have tested positive?	SPR - Canal 14
154	Are mental health factors also considered in the strategy you are implementing?	SPR - Canal 14
155	one of the symptoms is loss of smell diarrhea I do not know if this is true or not and I do not know if you could tell us	SPR - Canal 14
156	the new protocols at the airport	No identificado
157	If you can just remind us what the numbers of the hiring goal are for both doctors and nurses if they have a deadline to reach that level of recruitment	No identificado
158	if you have an estimate of how many resources in total are going to be spent in this recruitment process	No identificado
159	And taking advantage of how they are going to guarantee safety to doctors in certain areas, taking into account the high level of insecurity that exists in some regions?	No identificado
160	What would happen if the number of hires is not reached?	Reforma
161	Lethality of the states? CDMX is not the most lethal?	Reforma
162	Can influenza and COVID coincide?	Reforma
163	Could we get to the situation of the USA and Italy?	La Jornada
164	Can the fatality rate of Mexico be compared with the world? Are we approaching global lethality?	La Jornada
165	On hiring of health personnel. Are those who are going to stop working for their risk group included?	La Jornada
166	Regarding protective equipment, there is no homogeneity, and there are groups that feel at a disadvantage, what can you say about that?	AVA Noticias - Veracruz
167	With the supplies that will arrive, are the protests / complaints from health workers about the lack of supplies going to end?	AVA Noticias - Veracruz

168	In the mathematical model, how is the proportion infected today? is it more or is it less?	Glucosa Atómica
169	There are inmates without protection and with discrimination from the health authorities. Some expelled them and others received them contrary to what the Ministry of Health said. Is there incoordination? Is there already a unified position on this?	Glucosa Atómica
170	About the Polytechnic Transfer Factor. Gatell has said there is no evidence and a doctor is waiting for his approval. Do you plan to approve it?	Glucosa Atómica
171	Milenio talks about a hospital frightened by the virus. Can you check if it is true that it takes 5 days for patients to have their analytical results? Or clarify when they receive it?	Milenio
172	We have 6 to go to reach 100 deaths. Are they so high for the chronic diseases of the country? So in the most critical stage are more people going to die?	Milenio
173	They informed us of a policeman who was infected in VIVE Latino, is it real?	Milenio
174	What protocol will be followed for people with different abilities?	Diario Basta - Grupo Cantón
175	What procedures are carried out must be expressly followed by the capital security secretariat, well in this case the police corporations due to the case that today will be done today it has been announced that there are nine new infected by coronavirus and in fact this day yesterday that the police officer dies who was not known to have died from coronavirus	Diario Basta - Grupo Cantón
176	the treatment for people who die, what is it that has to be done?	La Jornada
177	What news do you have from Baja California today this information arrived that hospital services in that entity are already being exceeded by patients with coronavirus	La Jornada
178	The number of people who have died from this disease .... here, we only talk about the associated comorbidities. Are there several people who have died and do not have these diseases?	La Jornada
179	of the deaths, have they all occurred in a hospital? this has it is possible that some people have lost their lives outside the hospitals	La Jornada

180	on hydroxychloroquine, are there provisions to regulate the sale?	Sistema Público de Radiodifusión
181	how many private laboratories or private hospitals have been sanctioned for promoting or applying tests to detect COVID 19 without having the registry or complying with the INDRE regulations	Sistema Público de Radiodifusión
182	We update the data to date how many public hospitals and private laboratories can apply and have the use of INDRE	Sistema Público de Radiodifusión
183	It can be transmitted to animals as the protocol would change to avoid this type of transmission, and if in Mexico some surveillance is being carried out on the animal	El Universal
184	UNAM decided to withdraw its inmates from hospitals, and public health institutes, pointing out that there is no training for them and no conditions. If you could give us a comment concerning this	El Universal
185	This morning the president mentions that the support of Cuban doctors could be requested to support Mexico in this contingency if there is already a specific project or a formal request from the Mexican government and why make this request if there is a lack of specialists in Mexico	El Universal
186	There have already been threats in social networks, different news, but if there have already been direct physical attacks on the health personnel of the IMSS and also of the other institutions if Dr. Borja could tell us ...	Notimex TV
187	Since the metro is going to decrease the flow of trains, is there a contingency plan before phase 3 or for phase 3?	Notimex TV
188	people spit on the street and not only on the street but also in transportation, is this a means of contagion at this time of the pandemic?	Multimedios Televisión
189	For Dr. Víctor Hugo Borja I would like to ask him what is happening with the supplies of the doctors since even today we could see a protest precisely in the 76 hospital of the IMSS	Multimedios Televisión
190	I would like to ask you about the meeting you had a few days ago, which was a virtual meeting with governors with the chancellor and the Secretary of the Interior, if any approved measure was reached.	Multimedios Televisión

191	After April 30, what panorama can we expect, will activities resume or perhaps stricter measures will be taken?	Milenio
192	Dr. Borja, from this pandemic, how many consultations have received, how many disabilities have been generated from this digital tool, whether there is saturation or not?	Milenio
193	We learned about or documented an Oaxaca case of a delegate who escaped being positive for COVID and not only escaped but also spat on patients, spat on people for not giving preferential treatment. What can you tell us about it?	Milenio
194	These three people who are not part of the chronic people who do not have to because they died, that is, what was the factor that triggered what happened?	Canal once
195	ask him about the plane that is going to land at nine o'clock at night we have the exact schedule that brings the same is the amount that brings fans	Canal once
196	is that the only purchase to be made?	Canal once
197	This draft resolution that Mexico proposed to the UN, which proposed that there is effective access to medicines, equipment, and vaccines, this purchase that is made is within this framework, and if it is not, how will the proposal made by Mexico go?	Canal once
198	How many COVID hotels are there and how is their operation?	Notimex TV
199	There are six students from the National Polytechnic Institute and the Georgia Institute of Technology prototyping fans. I want to know if you guys are going to approach them?	Telemundo
200	Dr. Cortéz, do you already have a protocol for social confinement centers but now and the same also for other places where people such as migratory substances are confined?	Telemundo
201	That has already been detected cases of infection or diseases among the people who are confined?	Telemundo
202	with the known cases that exist so far in the country, what is the incidence rate, what is the secondary attack rate that has been detected, when, if so, serological screening could be done to review possible immunities	Telemundo

203	and also if there are plans to do post-mortem tests of cases that had not been confirmed before death	Telemundo
204	on the recent scientific literature that suggests that there is a significant percentage of infections in pre-symptomatic or asymptomatic cases, in that case, what would be the epidemiological response in the case of Mexico?	Sistema Público de Radiodifusión
205	Does the secretary of health have a registry of Mexicans residing abroad?	Sistema Público de Radiodifusión
206	Are there guidelines to repatriate bodies?	Excélsior
207	There is great concern in the IMSS hospitals in the states, such as regional 6 in Gómez Palacio, from where the samples are being sent to Mexico City, they take up to 5 days, in fact the journalist Javier Garza Ramos documented that 4 people died Before the results arrived, the question would be what percentage of the tests that are done in the country are being sent to Mexico?	Excélsior
208	... and how many days would it take on average to return?	Diario Basta - Grupo Cantón
209	what is the deficit of doctors if in October 2019 Zoé Robledo the head of the IMSS told us that there was a deficit, what would be the number of doctors or personnel that would be missing ...	Diario Basta - Grupo Cantón
210	How many patients in Mexico have been cured of the disease?	Círculo digital
211	It is said that the key emblematic works of the federal government are going to reactivate the steel cement industry only for the works of the federal government, does that seem correct to you? is it a measure according to contingency?	Círculo digital
212	Would it be more serious to stop the oven than to have it infected?	Círculo digital
213	mortality and sickness rate by age group ...	Uno TV
214	On the statement that it does not recommend using arches and sanitizing tunnels. What should the authorities do, remove the filters or that citizens do not pass?	Uno TV
215	How is the model paraphrased so that the population understands it?	Milenio
216	Is the number of infected 12% greater?	Milenio
217	What are these cases called? Estimates?	Milenio

218	Estimates must be in isolation?	Audiorama
219	On HIV / AIDS, they are closing services. Could Cases Rebound During the COVID Emergency? Does the secretariat have a position?	Audiorama
220	Were the numbers you presented estimated or are they going faster?	Medicina digital
221	From the graphs, we do not have access to the hospitalized, the number of patients ...	Medicina digital
222	There was a change in the technical statement. Why are 30 countries included and why before there were only 5	Reforma
223	Rapid tests will be delivered, is that part of the sentinel model?	Reforma
224	About the outbreaks in hospitals, what is happening there? Is it a lack of preparation, of material?	Grupo fórmula
225	The figures you have given on covid have given arguments to those who assure that they are playing with the figures. What is your opinion of it?	Grupo fórmula
226	Clinic 62 of the IMSS, which assures that there is an outbreak there and no material has reached them, in fact they protest contradicting the director Robledo. What can you say about that?	Notimex TV
227	Ask him about the 17 infected SEDENA commanders	Notimex TV
228	The head of government Claudia Sheinbaum calls the citizens of the capital to wear face masks, what would you say?	SDP-Noticias
229	Of this week's protocols, SERESO, if temporary releases are being contemplated?	SDP-Noticias
230	How is the discharge of a recovered person determined?	Pie de página
231	Pregnant women: what are the care we should have with those who are about to give birth?	Pie de página
232	If there is some type of blood that is needed more?	La Jornada
233	Have the cases of depression or mental health illnesses increased?	La Jornada
234	This new graph, disaggregated by age group and fatality rate, means that in Mexico more young people are getting infected but more older people are dying, if you could clarify this for us	Milenio
235	Today they make a specific call not to consume alcohol and we find out that the breweries are going	Milenio

	to start manufacturing this product. Do they contradict?	
236	What is being talked about in the health council about the cause of COVID. And what is Mexico doing about animal trafficking?	Notimex TV
237	How can you handle the population with autism that costs them the confinement? And the prisoners?	Notimex TV
238	Many people in the market, what is your opinion on that?	Plenilunia
239	In the graphs include the recovered and what is the situation with which they entered?	Plenilunia
240	There is confusion about factor 8, why is it not being applied with deaths?	Plenilunia
241	When does CPP show me off?	Sistema Público de Radiodifusión
242	On the attacks on the toilets: is the health ministry thinking of changing the strategy to make a call for this to stop?	Grupo fórmula
243	How many beds and fans are available?	Grupo fórmula
244	How many are hospitalizing a day?	Grupo fórmula
245	What information do the toilets give to the relatives of the patients? Why the anger? What is the mechanism of how to inform people?	No identificado
246	Do you have information about the death of a toilet in Tlatelolco? circulates on networks, is that a data breach for you?	No identificado
247	What is the state with the most fans?	No identificado
248	When the person dies without knowing that it was COVID, how does the death certificate come out?	Notimex TV
249	We are close to phase III. Would some areas of the city be completely fenced off?	Notimex TV
250	Today you met with INSABI, you can give us some information	Multimedios
251	On the networks they are saying: "Dr. Gatell was forced to reveal the truth." I would like to ask you to clarify this situation again.	No identificado
252	shouldn't it have a mic? ... I see you touching your cheek	No identificado
253	Due to the confinement, you do not know the possibility of implementing an online service so as not to leave these homes in distress?	No identificado

254	The thief's oil: eucalyptus, lemon, cinnamon. Is it valid that society can have this type of belief such as religious stamps?	Pie de página
255	How to talk to children about this?	Pie de página
256	Your projections are being fulfilled, are we going well or badly?	Notimex TV
257	Is it possible that AMLO will decree phase 3 tomorrow?	Milenio
258	AMLO asked the US government for the possibility of selling a hundred fans. What is the budget that was allocated. Are fans included in the China purchase?	Milenio
259	Within AMLO's request to politicians to donate their prerogatives, what is he talking about and the resources they need to face the emergency?	Milenio
260	How many people are intubated?	Excélsior
261	There is concern from people who have to go home with symptoms and are asked to stay for 14 days. Who diagnoses that they are no longer positive?	Excélsior
262	Some people get worse at home. What protocol must be followed? Does the family take them to the hospital or come to pick them up?	Excélsior
263	They have talked about the IMSS and the SS, but we have not heard data from the ISSSTE	Telemundo
264	Of those recovered, which have been extubations? Have sequelae been detected?	Telemundo
265	Of these, which are patients treated with plasma?	Telemundo
266	Of the essential workers, some already precarious, what suggestions do you have for those who distribute food, work in super or drive taxis to protect themselves and their clients?	La Jornada
267	Have you thought about changing the way you assess people with symptoms? Today I have known some cases of different people but if they only have chest pain to breathe. There is a person who died yesterday with these symptoms, and they did not do the test, nor did they receive him in the hospital, and he died yesterday.	La Jornada
268	How long on average are labs taking to get the result?	AVA Noticias - Veracruz
269	In networks, there has been a controversy over the supply of inputs with a comedian. Can you tell us about the flights you have scheduled and if there is a date for this?	AVA Noticias - Veracruz

270	The exact characteristics of the work risk bonus.	AVA Noticias - Veracruz
271	Is a voucher also being studied for the Ministry of Health?	AMPRYT
272	What do you recommend telling children? And if you recommend taking the children to work?	AMPRYT
273	Do you think you violated the childcare protocol by bringing your child to the national palace? You who are an example for Mexicans	Instituto Mexicano de la Radio
274	Has the ethical guide for deciding to prioritize medical care already been delivered to the medical staff? Is it already broadcast?	Pulso saludable
275	Is the speed of the spread of the virus because some people do not take these measures of healthy distance seriously, or is it the natural behavior of a pandemic like this one?	Pulso saludable
276	What do you think happens with the studies that already have a vaccine first or a treatment first?	Pie de página
277	several doctors who are against and medical personnel who are hired under a substitution regime this substitution is not earning 50% that the real bases do not have medical service or benefits and some are very worried because that is how they are going to do as they said yesterday the line frontal, not that it is going to be in the face of the epidemic, then what to do with them, how to give them attention, not if this is going to improve, yesterday a 20% bonus was announced to medical personnel, what happens with this other personnel that is left abandoned?	Pie de página
278	the guide for the management of corpses by COVID indicates that the final disposal of the corpse will be as soon as possible preferably by cremation then the diocesan center for human rights fray Juan de Larios issues a concern in a country in which there are 60 thousand disappeared and It is 30,000 people, unrecognized corpses, since corpses are created without being recognized, then, how can one make them have as their own identification?	Audiorama
279	You have told us about the economy, which is important, however, you have been asked to stop one of the most important industries, which is the brewery ... it was not classified as essential ... 800,000 small shops are being put at risk here where beer	MVS

	forms 40% of their income, the impact is also for 5000 farmers .. beyond not being a basic necessity, it is still a product of agroindustrial impact ... is this industry not being affected too much? is it needed?	
280	Today, you had a virtual meeting with the state secretaries. What are the agreements they reached with the issue of reconversion, especially with this monitoring of the use of medical services?	MVS
281	There is the possibility in this transition to phase 3 that not the whole country enters phase 3 at the same time, but rather the metropolitan area is the first to enter this phase ...	Milenio
282	Regarding the supplies, it has the details of in which states, if they were already delivered, was it confirmed that they were delivered and that they are already distributed in the clinics in which states has it been said that some said they were in this transfer process?	Milenio
283	what is contemplated in these flights?	Milenio
284	would there be possibilities that there could be more flights to guarantee supply?	Canal once
285	When can we also know the scientific evidence of people, for example, economists of sociologists, who tell us the pros and cons of this confinement?	La Jornada
286	speaking before the imminent arrival of phase 3 doctor that you have already alerted us for several days precisely what doctor Borja has just been, at what level, let's say, by saying a word about the occupation of hospital services, are we already? to have a clearer magnitude of what we are facing .. WHO has already talked about the level of contagion in LA, but we see that the worst is yet to come	La Jornada
287	Unfortunately, we have testimonies from people who have patients hospitalized with COVID that this telephone information system still does not work much. What can you tell us?	Canal once
288	Will there be an announcement tomorrow that we are in phase 3? or when will it be? will they make an announcement?	Canal once
289	What is the procedure or under what criteria are the supplies arriving on the planes being distributed?	Notimex TV

	which states first? Who should decide based on what criteria?	
290	About the national guard minutes before five in the afternoon because already on Twitter they launched that they are already taking to the streets in safety, will they make guards?	Notimex TV
291	Regarding supplies, there are hospitals that have held demonstrations yesterday, for example on October 1, today the hospital on November 20, they continue to complain that they do not provide supplies yet	Puntos suspensivos radio
292	José Antonio Lara Peinado, he left me a message in the book because he is working and the entire consultation is free by phone he is in Hidalgo, to help all cases of anxiety .. for work with children and others, I leave the data.	Reforma
293	If the total number of tests that have been carried out groups confirmed cases, negative cases and suspected cases?	Reforma
294	does the expansion factor change according to the entity? I tell you because on April 13 the head of government reported that there were 855 confirmed cases, that is, 6000 cases, understanding the sentinel model and well, it gives a multiplier of 7 ... which is different from the one that had been exposed	Reforma
295	in phase 3 how much could it grow?	Reforma
296	He said he was going to talk about the death estimate ...	MVS
297	He has had contact with some governors, especially from National Action, who through their social networks have reported that these supplies that they brought from medical personnel because it is of poor quality were there an error?	El Sol de México
298	Capital cities that you already speak of that are in a condition because well ahead of the rest of the country could we say since they are the red lights?	El Sol de México
299	Is there a confrontation with the governors over the quality of the supplies?	Notimex TV
300	You announced that the first cycle of the pandemic would be June 25, so what do you mean?	Notimex TV

301	In the morning you spoke something about segmenting the mobilization to the population that measure will be taken and if it is taken as it would be	Notimex TV
302	The governor of Michoacán today said that he is not going to take the recommendations into account. He asked me more about whether these states have the autonomy to decide whether or not to lift these measures.	No identificado
303	Since it was clarified that the bioethics guide is a project and now it specifies that it is going to evolve and is a living document, I would like to ask you, since from my point of view, it seems that it rests on a bio-political concept of making live letting die, in that sense if other philosophical perspectives could be included in this guide? if they are considering including other specialists? when will it be presented?	No identificado
304	If it is going to have legal precepts, how will the use of this guide be guaranteed to the ... to the first line of the doctors so that it becomes effective, how is it going to be disseminated, how is it going to be present for the people that goes on the front line ...	No identificado
305	and if some type of dialogue has already been established with the rector of the UNAM, who has left the guide	Rompeviento TV
306	Malnutrition is not considered a high risk disease against COVID 19? especially in relation to indigenous peoples.	Rompeviento TV
307	Are you, as the secretary of health, giving special attention to the displaced? In Chiapas, for example, there are people without a home.	Rompeviento TV
308	In this pandemic of fake news that irresponsibly launched by some journalists or the media, putting aside that part regarding the images that have been made of videos in hospitals or clinics that the logistical order in those clinics and hospitals looks a bit chaotic I ask if the Ministry of Health has or is preparing hospitals with a logistical order, how are people going to get there so that it is not chaos	MVS
309	About the bioethics guide, because I understand that the project and the guide are similar, the only thing that changes is that it will be put to the consultation	Diario Basta - Grupo Cantón

	of the members where I understand they would be secretaries of state and also the rector of the university of some presidents of medical associations What happens that happens first is discussed among all the members and then approved, can it still be modified according to the opinion of the members or is it made known to them when it is published in the DOF?	
310	On April 4, a newspaper sufficed announced the entry of four infected with coronavirus to the north prison, which was rejected with a note sent to the newsroom today thirteen days later, it is commented in various newscasts that a person dies from COVID. Why not act promptly?	Canal once
311	Municipalities to lift the quarantine ... how will this process be to lift this quarantine	La Jornada
312	in relation to the comment made by Governor Bonilla ... to what extent it is also a responsibility perhaps of the states because what we have understood is that these are deaths that have to be confirmed by laboratory	La Jornada
313	It is possible to know already what level of occupation they already have of the hospital facilities for cases or critics, that is, at what level they are already in 30 in 40	Pulso saludable
314	Is the population base you were talking about the answer to the fact that in some municipalities there are no null cases?	Pulso saludable
315	I have unfortunately witnessed acts of aggression against doctors where they can go to warn that this is happening and that it does not continue to occur so that it does not go unpunished	Pulso saludable
316	what would happen if you tremble with a patient who is in a timely manner	MVS
317	ask him if there is not a bond or something that has been broken or something that is not very useful between the communication between the authorities that take this issue of COVID 19 and the governors ... taking into account that they will have to arrive to more agreements like the ban on cremation of bodies.	MVS
318	You are going to abide by what the world health organization marks about re-counting the data the	Milenio

	figures you have on COVID 19 to prevent it from happening in cases like China	
319	about COVID-free municipalities, what are the criteria against which they are evaluated, why are they COVID-free?	Milenio
320	Regarding the fans, we know that an agreement was reached with the United States for the purchase of a thousand, how many would already be available	Milenio
321	A case of a youtuber who left decided to infect and right now it caused that there are already demands on the part of the population, is it possible to apply the law like this when a person deliberately leaves and can infect another person?	Instituto Mexicano de la Radio
322	Regarding the cremation agreement, just ask him if he has the data on how many of the deaths that have occurred so far	Instituto Mexicano de la Radio
323	how long it takes if you can specify a death that is suspicious to be confirmed by COVID 19	Instituto Mexicano de la Radio
324	According to a study, the State of Mexico, Veracruz and Chiapas appear to be the most vulnerable if you can tell me if they are concerned about the situation, especially in the State of Mexico	Audiorama
325	What about these asymptomatic cases, this I was reading an article where in China eight out of ten cases were asymptomatic and many related them to children, is this true? Is it feasible?	Audiorama
326	In the case of the regions, it has been contemplated to lift economic sanctions so that people who come from states that may be with transmission to others where not, have been contemplated?	AVA Noticias - Veracruz
327	A few days ago there was a note that said that the IMSS had bought some fans from a supplier in Guanajuato, which obviously has a branch in the United States but they had not been delivered, if you can tell us about that.	AVA Noticias - Veracruz
328	Yesterday an unpleasant event happened in a television host who spoke of you precisely about not taking the case from the health authority, not if you can give us your point of view	Excélsior
329	segob and ssalud, mentioned the term .. what does a warning mean?	Excélsior

330	Is each institution in charge of guaranteeing and reviewing the quality of the personal protective equipment of doctors?	Plenilunia
331	Would it be possible for us to have questions from other broadcasts?	Plenilunia
332	Regarding some data on lives saved, we can have an estimate of these actions how many lives they have allowed us to save	Plenilunia
333	is there any with the new COVID information if there are any new recommendations	Milenio
334	how many people have survived COVID and what is the follow-up given to observe the immunity	Milenio
335	is the situation of the children of divorced parents what happens in these cases when the children spend a week or fifteen days with one of the parents would run some risk in taking them and bringing them	Milenio
336	about the amnesty law if you can tell us	Milenio
337	You would see some risk that the 128 legislators are there, but it has been asked that they do not bring together more than 30 people.	Diario Basta - Grupo Cantón
338	These weeks the citizens were asked to wear face masks, especially in public transport, however, there are several people who bring a type of cloth, that is, instead of masks, even scarves, I do not know this, which is so feasible	Diario Basta - Grupo Cantón
339	how many people with HIV have died from COVID	Reforma
340	where do the fans come from	Reforma
341	If correct, these data for these 527 beds would be sufficient for stage 3	Doctor Politécnico
342	Have you considered inviting an official of the SEP to explain to us in greater detail about the school calendar	Doctor Politécnico
343	Now that the government did the same with this television station that they mentioned yesterday and you have considered as a health authority to file charges or some action why they discredited them	Heraldo de México
344	Because no further measures have been taken with nearly 10,000 CAPUFE workers who are still operating the country booths and so far there are 21 suspected cases, and they are afraid.	Heraldo de México
345	Between now and May 8, the most critical scenario is expected in the Valley of Mexico Mexico City and the	Multimedios Televisión

	state of Mexico. How many beds for intensive care for critical care?	
346	Yesterday in our midst, the death of a man by COVID 19 was documented by the municipality Miguel Hidalgo. This man began with mild symptoms, and he was not followed up as a suspected case ... my question is, do you have a record of how many patients are dying? in their homes?	Multimedios Televisión
347	There is a protocol precisely for when these deaths are carried out in people's homes	Pie de página
348	is there a strategy to attend nursing homes ?, pregnant women, migrants	Notimex TV
349	some companies asked their workers to come back today ...	Notimex TV
350	How is the medical recruitment day going? Some young nurses from the Chiapas area there in Juchitán, well, they mention that they attended the recruitment but that they have not been called what is happening	El Universal
351	on community outbreaks ... if these entities will be where the first measures of phase 3 of the contingency will be dictated	El Universal
352	how close we are to getting to phase 3	El Universal
353	people who want to say goodbye to their relatives, is there any protocol to say goodbye?	Canal once
354	about this meeting that was held at 5 in the afternoon the general health council that can tell us in what tenor what are the issues that were discussed	Diario Basta - Grupo Cantón
355	Question for mtra. Fabiana, you don't think there should be more severe laws that punish those who attack medical personnel	Multimedios Televisión
